# The use of mosquito repellents at three sites in India with declining malaria transmission: surveys in the community and clinic

**DOI:** 10.1186/s13071-016-1709-9

**Published:** 2016-07-27

**Authors:** Anna Maria van Eijk, Lalitha Ramanathapuram, Patrick L. Sutton, Nandini Peddy, Sandhya Choubey, Stuti Mohanty, Aswin Asokan, Sangamithra Ravishankaran, G Sri Lakshmi Priya, Justin Amala Johnson, Sangeetha Velayutham, Deena Kanagaraj, Ankita Patel, Nisha Desai, Nikunj Tandel, Steven A. Sullivan, Samuel C. Wassmer, Ranveer Singh, K Pradhan, Jane M. Carlton, H. C. Srivasatava, Alex Eapen, S. K. Sharma

**Affiliations:** 1 Center for Genomics and Systems Biology, Department of Biology, New York University, New York, NY 10003 USA; 2 Jigyansha, International Center of Excellence for Malaria Research, Sector 1, Raurkela, Odisha India; 3 National Institute of Malaria Research Field Unit, Indian Council of Medical Research, National Institute of Epidemiology Campus, Ayapakkam, Chennai, Tamil Nadu India; 4 National Institute of Malaria Research Field Unit, Civil Hospital, Nadiad, Gujarat India; 5 London School of Hygiene and Tropical Medicine, Keppel St, London, WC1E 7HT UK; 6 National Institute of Malaria Research, Indian Council of Medical Research, Dwarka Sector 8, New Delhi, India; 7 Acsel Health, 500 5th Ave, Suite 2760, New York, NY 10110 USA

**Keywords:** Mosquito control, Repellents, Urban, Rural, Education, Socio-economic status, *Plasmodium falciparum*, *Plasmodium vivax*

## Abstract

**Background:**

Repellents such as coils, vaporizers, mats and creams can be used to reduce the risk of malaria and other infectious diseases. Although evidence for their effectiveness is limited, they are advertised as providing an additional approach to mosquito control in combination with other strategies, e.g. insecticide-treated nets. We examined the use of repellents in India in an urban setting in Chennai (mainly *Plasmodium vivax* malaria), a peri-urban setting in Nadiad (both *P. vivax* and *P. falciparum* malaria), and a more rural setting in Raurkela (mainly *P. falciparum* malaria).

**Methods:**

The use of repellents was examined at the household level during a census, and at the individual level in cross-sectional surveys and among patients visiting a clinic with fever or other symptoms. Factors associated with their use were examined in a multivariate analysis, and the association between malaria and the use of repellents was assessed among survey- and clinic participants.

**Results:**

Characteristics of participants differed by region, with more people of higher education present in Chennai. Use of repellents varied between 56–77 % at the household level and between 32–78 % at the individual level. Vaporizers were the main repellents used in Chennai, whereas coils were more common in Nadiad and Raurkela. In Chennai and Nadiad, vaporizers were more likely to be used in households with young male children. Vaporizer use was associated with higher socio-economic status (SES) in households in Chennai and Nadiad, whereas use of coils was greater in the lower SES strata. In Raurkela, there was a higher use of coils among the higher SES strata. Education was associated with the use of a repellent among survey participants in Chennai and clinic study participants in Chennai and Nadiad. Repellent use was associated with less malaria in the clinic study in Chennai and Raurkela, but not in the surveys, with the exception of the use of coils in Nadiad.

**Conclusions:**

Repellents are widely used in India. Their use is influenced by the level of education and SES. Information on effectiveness and guidance on choices may improve rational use.

**Electronic supplementary material:**

The online version of this article (doi:10.1186/s13071-016-1709-9) contains supplementary material, which is available to authorized users.

## Background

Vector-borne diseases are a considerable burden in India. Malaria was estimated to account for approximately 17 million episodes of illness and 26,000 deaths in India in 2013 [1]. Dengue epidemics are on the increase in India, and resulted in about 5.8 million cases per year between 2006 and 2012 [2]. In certain areas in India, other vector borne diseases such as chikungunya, lymphatic filariasis and Japanese encephalitis are of great concern [3–5]. Use of protection against mosquito bites may result in prevention of these diseases in addition to reducing mosquito annoyance and itching. For malaria control, insecticide-treated nets (ITNs) and indoor residual spraying are methods with proven efficacy, commonly used in elimination efforts [6–9]. In India, China and other countries, there is an extensive internal market for additional mosquito repellents providing personal protection; these include coils, vaporizers, mats and creams (Table 1). They generally work by inhibiting the olfactory receptors of mosquitoes, which interfere with their host-seeking behavior [10, 11]. Whereas coils can be used anywhere, vaporizers and mats typically require electricity. Repellent creams have the advantage of providing “mobile protection”; their ingredients vary, including N,N-Diethyl-meta-toluamide (DEET) and Picaridin [12, 13]. Repellent use is widespread in India, regularly surpassing net use (Additional file 1: Table S1) [14–19], and at considerable costs to households (0.6–3.0 % of per capita income) [14, 15]. Compared to insecticide treated nets or indoor residual spraying, the efficacy of these personal protection methods has not been widely evaluated [6, 7]. Studies to evaluate the epidemiology of malaria at three sites in India (Chennai, Nadiad and Raurkela), as part of the Center for the Study of Complex Malaria in India [20], provided an opportunity to assess which personal protection methods were used, who was using them, and if an association between their use and malaria could be detected in the respective regions.

**Table 1:** Description of mosquito repellents

Repellent	Description
Coil	Mixture of repellent powder and a combustible filling material and a binder in the shape of a coil. Releases repellent in the air when burning
Vaporizer (dispensers)	A reservoir bottle with fluid repellent which is evaporated by an electric heater through a porous wick
Mat	Pads impregnated with a volatile repellent which needs to be heated on a small electric heating plate to vaporize
Cream	Skin cream or oil supplemented with a repellent
Emanator	Special absorbent material which slowly evaporates a suitable repellent at room temperature (strips or small box)

Adapted from [57]

## Methods

### Settings

**Fig. 1:**
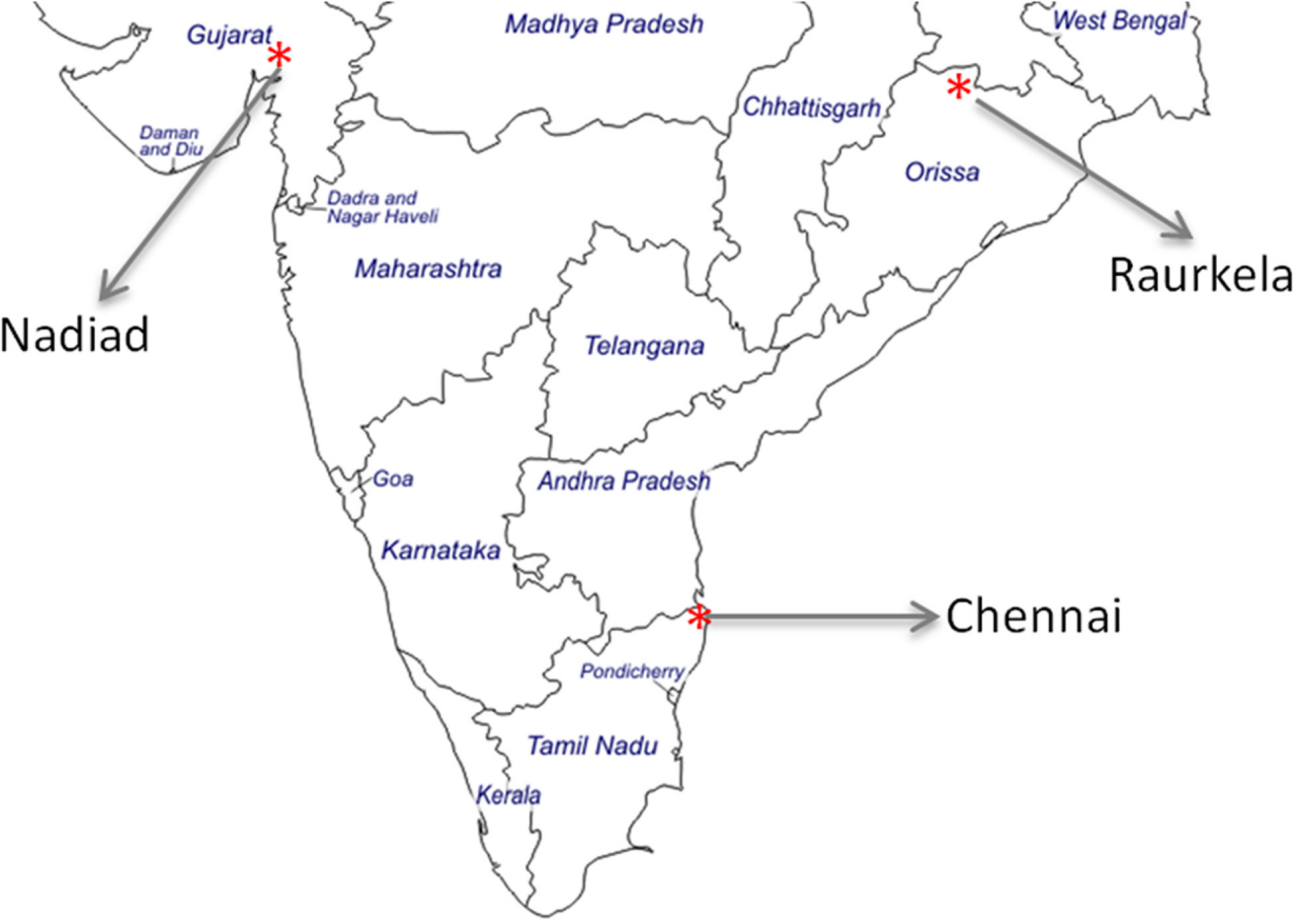
Map of study sites in India, 2012–2015

Chennai, the capital of the southern state of Tamil Nadu, is located on the coast of the Bay of Bengal (Fig. 1) and has a population of ~4.7 million and population density of 26,903/km^2^ according to the most recent census in 2011 [21]. The climate in Chennai is categorized as ‘tropical wet and dry’, with temperatures ranging from ~15 (January) to ~45 °C (May) and a relative humidity between 59 and 80 %. Monsoons come in two waves; the main rainfall period is from October–December as part of the northeast monsoon, but some rains also come during the southwest monsoon in July-August [22]. Malaria transmission (predominantly *Plasmodium vivax*) in Chennai city is perennial and peaks between July and October; the main malaria vector is *Anopheles stephensi* [23, 24]. Census, cross-sectional surveys, and clinic studies were conducted in the catchment area and at the Besant Nagar clinic, part of the Regional Office of Health and Family Welfare of the Government of India in a predominantly residential neighborhood in Chennai composed of middle and upper class dwellings, with a few slums and a large coastal fishing community.

Nadiad town is located in the Kheda district in the central part of Gujarat State (Fig. 1) and has a population of ~225,132. It has a sub-tropical and semi-arid climate, receiving the majority of its annual precipitation during the southwest monsoon season (June–September) [22]. Malaria is considered to be hypo-endemic, with *P. vivax* and *P. falciparum* prevalence rates oscillating throughout the year based on the transmission season; the main malaria vector is *An. culicifacies* [23, 24]. The National Institute of Malaria Research (NIMR) Malaria Clinic is located in the Civil Hospital at Nadiad in a predominantly residential neighborhood. Census was conducted in the residential areas around the NIMR malaria clinic and in Sevaliya and Chetarsumba, two rural areas of the Kheda district close to Nadiad town with higher reported malaria endemicity.

Raurkela city is located in the Sundargarh district of the eastern state of Odisha (Fig. 1) and has a population of ~552,970 [21]. Raurkela has a ‘tropical wet and dry’ climate receiving heavy rains during the southwest monsoon season (June-September) and some rainfall during the retreating northeast monsoon (December-January) [22]. Malaria displays meso- to hyper-endemic transmission in this region, with *P. falciparum* as the major infecting species in the district; the main malaria vectors are *An. fluviatilis* and *An. culicifacies* [23, 24]. A new malaria clinic and research laboratory was developed at the Health Centre in Sector 1 due to its proximity to nearby slum areas. Census was conducted in the areas around Sector 1 Health Centre and the rural forested areas of Sundargarh district with higher reported malaria endemicity.

### Procedures

At each site, census information was collected by household on type of housing, water supply, use of malaria protection and demographics of the household members (age, gender, education and occupation). Four cross-sectional surveys were conducted over two years, each drawing subjects from a different random selection of census household members. After obtaining consent from the participant, they were interviewed using a structured questionnaire with sections on malaria history and use of mosquito protection; blood was also collected for microscopy and hemoglobin assessment. To increase the chance of detecting malaria, the surveys in Nadiad and Raurkela focused on rural segments of the census area. Patients visiting the malaria clinics at the three sites with symptoms indicative of malaria were enrolled in the clinic-based study and subjected to the same questionnaire and blood tests as used in the community survey, but were not linked to census data.

### Laboratory tests

Laboratory tests were performed for all individuals enrolled in the cross-sectional surveys and clinic studies. Hemoglobin level was assessed at the time of enrollment using HemoCue (HemoCue, Ängelholm, Sweden). Thin and thick smears obtained from blood collected via a finger prick were stained using Giemsa and at least 300 fields in the thick smear were examined using the 100× oil immersion before a slide was determined negative for malaria. Parasites were counted on the thick smear against 200–500 white blood cells (WBCs). The results were expressed as parasites per microliter of blood, using the WBC count if known, or assuming 8,000 WBCs per microliter of blood. Slides were routinely read by two microscopists and a third was used if there was disagreement. In addition, 10 % of the slides were re-checked for quality control.

### Analysis

A secure, web-based REDCap (Research Electronic Data Capture) database was used to capture and store all participant data and test results [25]. Subsequently, data were exported into Stata (Stata/IC version 13.1, StataCorp LP, College Station, USA) for analysis. For the census, which was conducted at the household level, principle components analysis was used to construct a wealth index to assess the effect of socio-economic status (SES) [26, 27], whereby the following household characteristics were used: building material of the house, source of drinking water, household ownership, electricity, livestock, education and occupation of the head of the household and routine source of health care (private, public or a combination). For all data sets, only households and individuals with information available on repellents were included in the analysis. For each site, repellents were defined as the use of coils, vaporizers, mats or creams; determinants of overall use of repellents and the two most common types used in a region were examined. Using census data, the following factors were evaluated for their association with the use of repellents at the household level: presence of a young (< 5 years of age) male or female child in the household; gender of the head of the household; presence of at least one person with secondary education in the household; SES; presence of at least one household member with an episode of malaria within the last year; season of interview and (for Raurkela only) ITN use. The following factors were examined at the individual level in the surveys: gender, age, education, a history of a malaria episode in the last year, season of interview, SES of the household and (for Raurkela only) ITN use. For clinic participants the same individual level factors were examined, except that occupation of the participant replaced SES. In the survey and clinic data the association between the use of repellent and malaria, and of repellent and anemia were explored. Generalized linear regression with a log link and binomial distribution was used for multivariate analyses, and Poisson regression with a robust variance estimator was used for models which did not converge [28]. Factors with a *P*-value < 0.1 in the univariate model were included in the multivariate model, and factors with a *P*-value > 0.05 were removed from the model, except for factors of special interest (e.g. young female child if young male child was significant in the multivariate model). The multivariate model for surveys was adjusted for clustering at the household level.

## Results

### Study characteristics

The studies were conducted between January 2012 and April 2015. In Nadiad and Raurkela, the surveys were conducted in the rural segment of the census area, resulting in some differences in characteristics between the census households and the households included in the surveys, e.g. the latter were poorer and had less educated household members (Additional file 1: Table S2). There were multiple differences in characteristics of households in urban Chennai, peri-urban Nadiad and rural Raurkela (Table 2). Almost all households had access to electricity in Chennai and Nadiad, whereas approximately 19 % of households were without electricity in Raurkela. The number of households with young children was higher in Raurkela (*χ*^*2*^ = 105.86, *df* = 2, *P* < 0.001 compared to other sites), whereas in Chennai the levels of education and salaried employment were higher. In Raurkela, 21.6 % of the households had one or more members with a history of malaria in the past year, compared with 16 % in Chennai and 8 % in Nadiad (Table 2). Household interviews were more likely to be conducted in the rainy season in Chennai than in the other sites. Characteristics were also different when comparing survey and clinic study populations, with a significantly higher proportion of males in the clinic population compared to survey participants across all sites (Table 3). Clinic participants were more educated (Nadiad and Raurkela) and more likely to have salaried employment (all sites) than the cross-sectional survey participants (Table 3).

**Table 2:** Characteristics of households participating in census at three sites in India, 2012–2014

	Chennai (*n* = 1,483)	Nadiad (*n* = 1,832)	Raurkela (*n* = 1,204)	*χ* ^2^, *P*-value (*df* = 2)
Time period of data-collection	Jan 2012–Oct 2014	Nov 2012–Oct 2014	Apr 2012–Apr 2014	
Electricity in household (%)	1,479 (99.7)	1,764 (96.3)	975 (81.0)	418.87, < 0.001
Livestock (%)	28/1,464 (1.9)	217 (11.8)	699 (58.1)	1,400, < 0.001
Average number of people in the household (SD), range	4.2 (1.6), 1–21	5.2 (2.5), 1–16	5.2 (2.1), 1–24	ANOVA: *F*_(2, 4515)_ = 100.53, *P* < 0.001
Child < 5 years in household (%)	374 (25.2)	463 (25.3)	494 (41.0)	105.86, < 0.001
Young male child (%)	215 (14.5)	277 (15.1)	305 (25.3)	67.13, < 0.001
Young female child (%)	206 (13.9)	255 (13.9)	287 (23.8)	63.06, < 0.001
Male head of household (%)	1,270/1,457 (87.2)	1,504/1,764 (85.3)	1,051 (87.3)	3.49, 0.174
Head of household salaried employment (%)	543/1,433 (37.9)	335/1,764 (19.0)	1,20/1,202 (10.0)	313.23, < 0.001
Head of household at least secondary education	553/1,217 (45.4)	441/1,764 (25.0)	53 (4.4)	543.35, < 0.001
≥ 1 person with at least secondary education in household (%)	971 (65.5)	945 (51.6)	301 (25.0)	443.46, < 0.001
Head of household had malaria in previous year (%)	94/1,460 (6.4)	55/1,764 (3.1)	132/1,202 (11.0)	74.38, < 0.001
≥ 1 person with malaria in the previous year (%)	237 (16.0)	146 (8.0)	260 (21.6)	116.07, < 0.001
1 person	187 (12.6)	121 (6.6)	139 (11.5)	53.94, < 0.001
2 persons	33 (2.2)	18 (1.0)	82 (6.8)	(*df* = 4)
> 2 persons	17 (1.2)	7 (0.4)	39 (3.2)	
Interview in rainy season (%)	462 (31.2)	109 (6.0)	182 (15.1)	377.72, < 0.001

**Table 3:** Characteristics of study participants by location and type of study at three sites in India, 2012–2015

	Cross-sectional study			Clinic study		
	Chennai (*n* = 928)	Nadiad (*n* = 796)	Raurkela (*n* = 1,539)	Chennai (*n* = 1,054)	Nadiad (*n* = 685)	Raurkela (*n* = 1,875)
Time period	Dec 12–Oct 14	May 13–Sep 14	Jan 13–Sep 14	Apr 12–Mar 15	Jan 13–Apr 15	Apr 12–Apr 15
Mean age, *n* (95 % CI, years)	33.3, *n* = 927, (32.3–34.3)^a^	34.2, *n* = 796, (32.9–35.5)	28.4, *n* = 1539, (27.5–29.3)	31.8, *n* = 1054, (31.0–32.6)^b^	27.4, *n* = 685, (26.1–28.7)	29.4 *n* = 1875, (28.5–30.3)
Age						
< 5 years, *n* (%)	22 (2.4)^c^	20 (2.5)	157 (10.2)	10 (1.0)^d^	26 (3.8)	162 (8.6)
5–9 years, *n* (%)	48 (5.2)	57 (7.2)	181 (11.8)	28 (2.7)	89 (13.0)	184 (9.8)
10–17 years, *n* (%)	76 (8.2)	90 (11.3)	186 (12.1)	106 (10.1)	110 (16.1)	277 (14.8)
> 17 years, *n* (%)	781 (84.3)	629 (79.0)	1,015 (66.0)	910 (86.3)	460 (67.2)	1,252 (66.8)
Male, *n* (%)	359 (38.7)^e^	379 (47.6)	692 (45.0)	717 (68.0)^f^	414 (60.4)	1,076 (57.4)
Among persons ≥ 18 years	*n* = 782	*n* = 629	*n* = 1,015	*n* = 910	*n* = 460	*n* = 1,252
Highest level of education secondary or higher, *n* (%)	472 (60.4)^g^	126/628 (20.1)	60 (5.9)	569/907 (62.7)^h^	124 (27.0)	683 (54.6)
Salaried employment *n* (%)	195/780 (25.0)^i^	32/627 (5.1)	20 (2.0)	480/904 (53.1)^j^	90 (19.6)	387 (30.9)

^a^ANOVA: *F*_(2, 3259)_ = 36.20, *P* < 0.001. ^b^ANOVA: *F*_(2, 3611)_ = 14.13, *P* < 0.001. ^c^ *χ* ^*2*^ = 149.55, *df* = 6, *P* < 0.001. ^d^ *χ* ^*2*^ = 194.40, *df* = 6, *P* < 0.001. ^e^ *χ* ^*2*^ = 15.41, *df* = 2, *P* < 0.001. ^f^ *χ* ^*2*^ = 32.27, *df* = 2, *P* < 0.001. ^g^ *χ* ^*2*^ = 683.69, *df* = 2, *P* < 0.001. ^h^ *χ* ^*2*^ = 160.58, *df* = 1, *P* < 0.001. ^i^ *χ* ^*2*^ = 279.40, *df* = 2, *P* < 0.001. ^j^ *χ* ^*2*^ = 181.06, *df* = 1, *P* < 0.001

### Use of personal mosquito protection

**Fig. 2:**
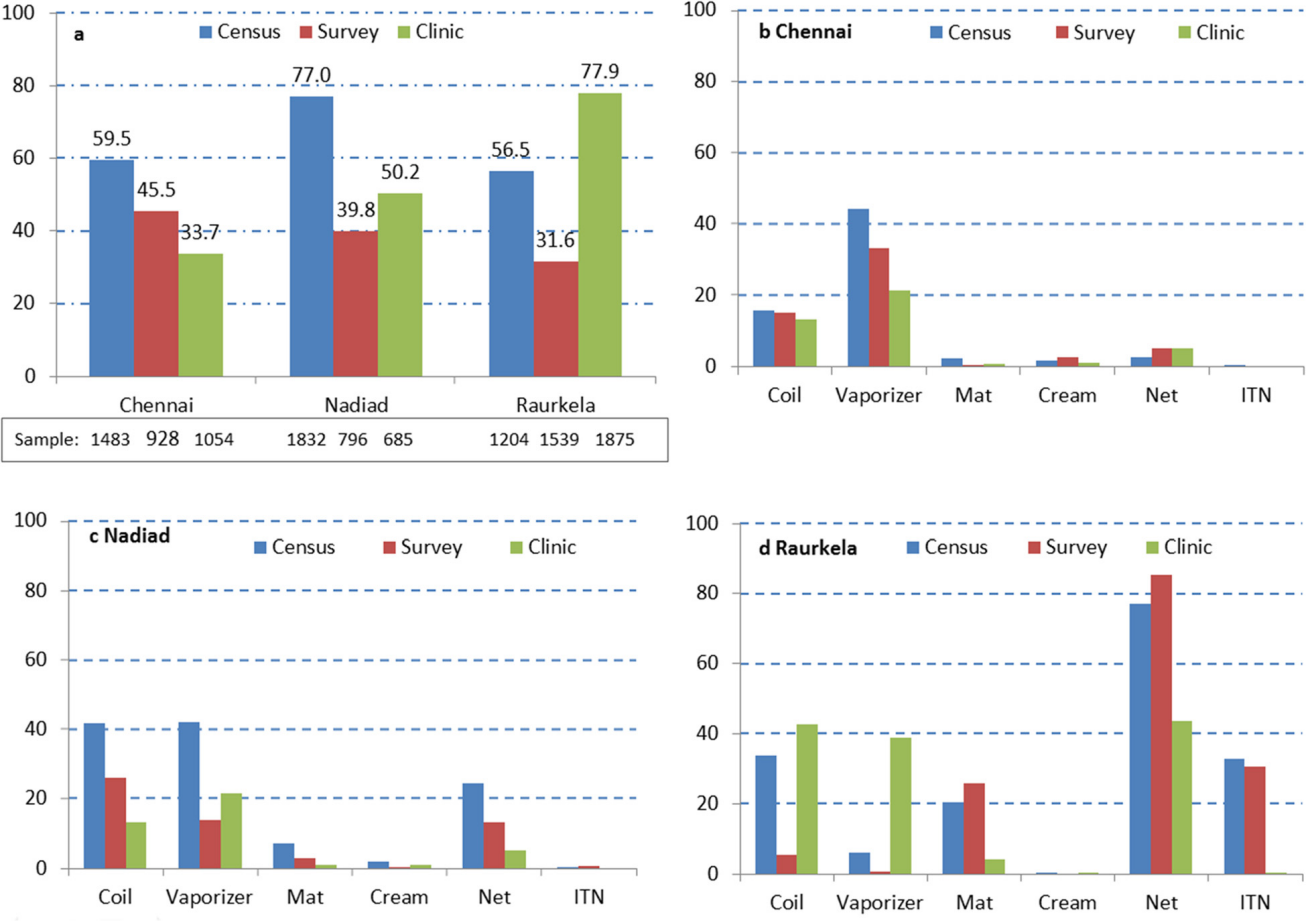
Use of personal malaria protection at three sites in India, 2012–2015. **a** Use of any repellent (percentage: coils, mats, vaporizers or creams. Use of repellent by region (percentage) and type of study: **b** Chennai, **c** Nadiad, **d** Raurkela. Note that the census assessed use of repellents at the household level and not at the individual level; the survey and the clinic assessed use at the individual level. The data for this graph are available in the Additional file 1: Table S3

The reported use of repellents was higher at the household level than by individuals in the surveys or the clinic studies except for Raurkela, where the use of repellents was higher in the clinic study compared to the households (Fig. 2, Additional file 1: Table S3). Vaporizers predominated in Chennai (44.2, 33.4 and 21.5 % in census, survey and clinic, respectively), whereas coils were more common at the other two sites (Nadiad: 41.9 %, 26.1 % and 27.7 %; Raurkela: 33.6 %, 5.4 % and 42.6 %). The overall use of creams was low (< 3 %) and mats were mainly reported in Raurkela (20.3 %, 25.7 % and 4.2 % in census, survey and clinic, respectively). The use of ITNs was low, except in Raurkela where 42.5 % households and 35.7 % individuals reported ITN use in the census and surveys.

### Factors associated with the use of mosquito repellents

#### Census: household level

In Chennai, repellents were more likely to be used in households with young boys, with higher educated household members, with higher SES and during the rainy season (Table 4, Additional file 1: Table S4A). Vaporizers were more common and coils less common in higher SES households (Table 4). Repellents were also more common in higher SES households in Nadiad, but were less likely to be used during the rainy compared to the dry season (Additional file 1: Table S4B). In Raurkela, the use of coils was associated with higher SES and a female head of the household; coils were used less in households with ITNs and less during the rainy season, whereas mats or a combination of repellents were more common in households with ITNs (Table 4). Households in Raurkela with members who suffered malaria in the past year were less likely to use repellents. Reported ITN use in Raurkela was associated with rainy season and the use of mats, and was less in household with a higher SES (Additional file 1: Table S4C).

**Table 4:** Factors associated with the report of the use of a repellent at the household level in multivariate analysis

	Any repellent		Vaporizer		Coils	
	Risk ratio (95 % CI)	*P*	Risk ratio (95 % CI)	*P*	Risk ratio (95 % CI)	*P*
Chennai Census						
Female < 5 years in HH	1.05 (0.93–1.18)	0.455	1.12 (0.96–1.30)	0.138	ns	
Male < 5 years in HH	1.17 (1.05–1.29)	0.003	1.22 (1.06–1.40)	0.006	ns	
At least one person with secondary education in HH	1.26 (1.12–1.41)	< 0.001	1.38 (1.18–1.60)	< 0.001	ns	
Male head of HH	ns		1.27 (1.02–1.58)	0.035	0.63 (0.48–0.82)	0.001
Socio-economic status						
High	1.37 (1.18–1.59)	< 0.001	2.22 (1.75–2.81)	< 0.001	0.28 (0.17–0.47)	< 0.001
2	1.32 (1.14–1.53)	< 0.001	2.17 (1.70–2.73)	< 0.001	0.52 (0.36–0.74)	< 0.001
3	1.06 (0.90–1.24)	0.487	1.46 (1.13–1.89)	0.004	0.54 (0.39–0.76)	< 0.001
4	1.13 (0.97–1.32)	0.110	1.55 (1.20–2.00)	0.001	0.73 (0.54–0.98)	0.037
Low	Reference				Reference	
Rainy season	1.33 (1.21–1.46)	< 0.001	1.14 (1.01–1.30)	0.038	2.13 (1.68–2.70)	< 0.001
Nadiad Census						
Female < 5 years in HH	1.12 (1.05–1.19)	< 0.001	0.95 (0.82–1.10)	0.486	1.24 (1.09–1.41)	0.001
Male < 5 years in HH	1.09 (1.03–1.15)	0.005	1.14 (1.00–1.30)	0.045	1.18 (1.04–1.34)	0.010
Person with secondary education in HH	ns		1.48 (1.29–1.70)	< 0.001	0.81 (0.71–0.92)	0.001
Socio-economic status						
High	1.93 (1.72–2.17)	< 0.001	16.63 (9.17–30.14)	< 0.001	0.88 (0.71–1.08)	0.223
2	1.93 (1.72–2.17)	< 0.001	15.25 (8.41–27.62)	< 0.001	1.04 (0.86–1.26)	0.679
3	1.82 (1.62–2.05)	< 0.001	12.18 (6.71–22.12)	< 0.001	1.19 (1.01–1.40)	0.041
4	1.43 (1.26–1.63)	< 0.001	5.37 (2.90–9.98)	< 0.001	1.26 (1.08–1.47)	0.004
Low	Reference		Reference		Reference	
Rainy season	0.46 (0.34–0.61)	< 0.001	0.36 (0.18–0.70)	0.003	0.44 (0.37–0.48)	< 0.001
Raurkela Census						
Male head of household	0.87 (0.78–0.97)	0.013	0.69 (0.50–0.95)	0.022	0.88 (0.79–0.99)	0.037
Socio-economic status						
High	1.89 (1.59–2.24)	< 0.001	0.02 (0.00–0.12)	< 0.001	47.96 (12.12–189.71)	< 0.001
2	1.67 (1.41–1.97)	< 0.001	0.10 (0.05–0.22)	< 0.001	45.21 (11.41–179.21)	< 0.001
3	0.65 (0.52–0.81)	< 0.001	0.41 (0.31–0.55)	< 0.001	11.36 (2.75–46.95)	0.001
4	0.74 (0.62–0.88)	0.001	0.69 (0.57–0.83)	< 0.001	2.24 (0.47–10.57)	0.309
Low	Reference		Reference		Reference	
Member(s) with a history of malaria last year	0.63 (0.53–0.75)	< 0.001	ns		0.68 (0.52–0.90)	0.007
Rainy season	ns		ns		0.54 (0.35–0.83)	0.005
ITN use	1.66 (1.45–1.90)	< 0.001	4.20, 3.15–5.59	<0.001	0.71 (0.55–0.92)	0.010

*Abbreviations*: *HH*, household; *ITN*, insecticide treated net; *ns*, not significant

Factors examined: young male or female in the household, person with secondary education in household, gender of the household, socio-economic status, a household member with a history of fever in the past year and season of interview. For tables with univariate and multivariate analyses, see Additional file 1: Table S4A–C

#### Survey: individual level

Findings were similar in the survey data with regards to SES (Table 5, Additional file 1: Table S5A–C). An individual with at least secondary education was more likely to use repellents in Chennai and coils in Raurkela. Survey participants were more likely to report coil use in the rainy season in Raurkela, as a contrast to the census information where households in Raurkela were less likely to report the use of coil in the rainy season.

**Table 5:** Factors associated with the report of personal malaria protection in surveys (individual level)

	Any repellent		Vaporizer		Coil	
	Risk ratio (95 % CI)	*P*	Risk ratio (95 % CI)	*P*	Risk ratio (95 % CI)	*P*
Chennai Survey						
At least Secondary education	1.28 (1.09–1.50)	0.002	ns		ns	
Socio-economic status						
High	ns		1.62 (0.90–2.91)	0.106	0.12 (0.04–0.40)	0.001
2			1.79 (1.04–3.09)	0.036	0.59 (0.30–1.16)	0.126
3			1.81 (1.05–3.13)	0.034	0.58 (0.30–1.13)	0.110
4			1.75 (1.02–3.02)	0.043	0.92 (0.52–1.64)	0.775
Low			Reference		Reference	
Nadiad Survey						
Socio-economic status						
High	4.45 (2.90–6.82)	< 0.001	15.83 (5.68–44.13)	< 0.001	4.18 (2.13–8.21)	< 0.001
2	4.30 (2.82–6.56)	< 0.001	20.00 (7.43–53.87)	< 0.001	3.73 (1.87–7.43)	< 0.001
3	3.13 (1.93–5.08)	< 0.001	6.75 (1.97–23.20)	0.002	3.80 (1.91–7.56)	< 0.001
4	1.33 (0.76–2.34)	0.315	3.22 (1.03–10.00)	0.044	1.90 (0.91–3.96)	0.087
Low	Reference		Reference		Reference	
Raurkela Survey						
At least secondary education	ns		0.40, 0.20–0.81	0.010	2.08, 0.96–4.51	0.064
Socio-economic status						
High	2.41 (1.46–3.97)	0.001	ns		21.95 (8.12–59.32)	< 0.001
2	1.03 (0.45–2.37)	0.945			5.18 (1.40–19.16)	0.014
3	0.83 (0.58–1.19)	0.316			1.61 (0.59–4.37)	0.348
4	0.79 (0.61–1.03)	0.087			0.95 (0.38–2.40)	0.915
Low	Reference		Reference		Reference	
Rainy season	ns		ns		3.27 (1.55–6.89)	0.002
ITN use	2.71 (2.14–3.44)	< 0.001	3.79 (2.91–4.92)	< 0.001	0.20 (0.06–0.66)	0.008

*Abbreviations*: ITN, insecticide treated net; NS, not significant

Note: multivariate analysis adjusted for clustering at the household level

For tables with univariate analyses see Additional file 1: Tables S5a–c

#### Clinic: individual level

Among clinic participants in Chennai and Nadiad, use of repellents was higher among people with at least secondary education, whereas at all three sites repellent use was significantly less common among workers with a daily wage compared to workers with a regular salary (Table 5, Additional file 1: Table S6A–C). In Nadiad and Raurkela, variable age groups were associated with vaporizer or coil use. In Chennai and Raurkela, vaporizers were less likely to be used during the rainy season, whereas in Nadiad they were more likely to be used in the rainy season among the clinic study participants.

### Association between the use of personal malaria protection and malaria

**Fig. 3:**
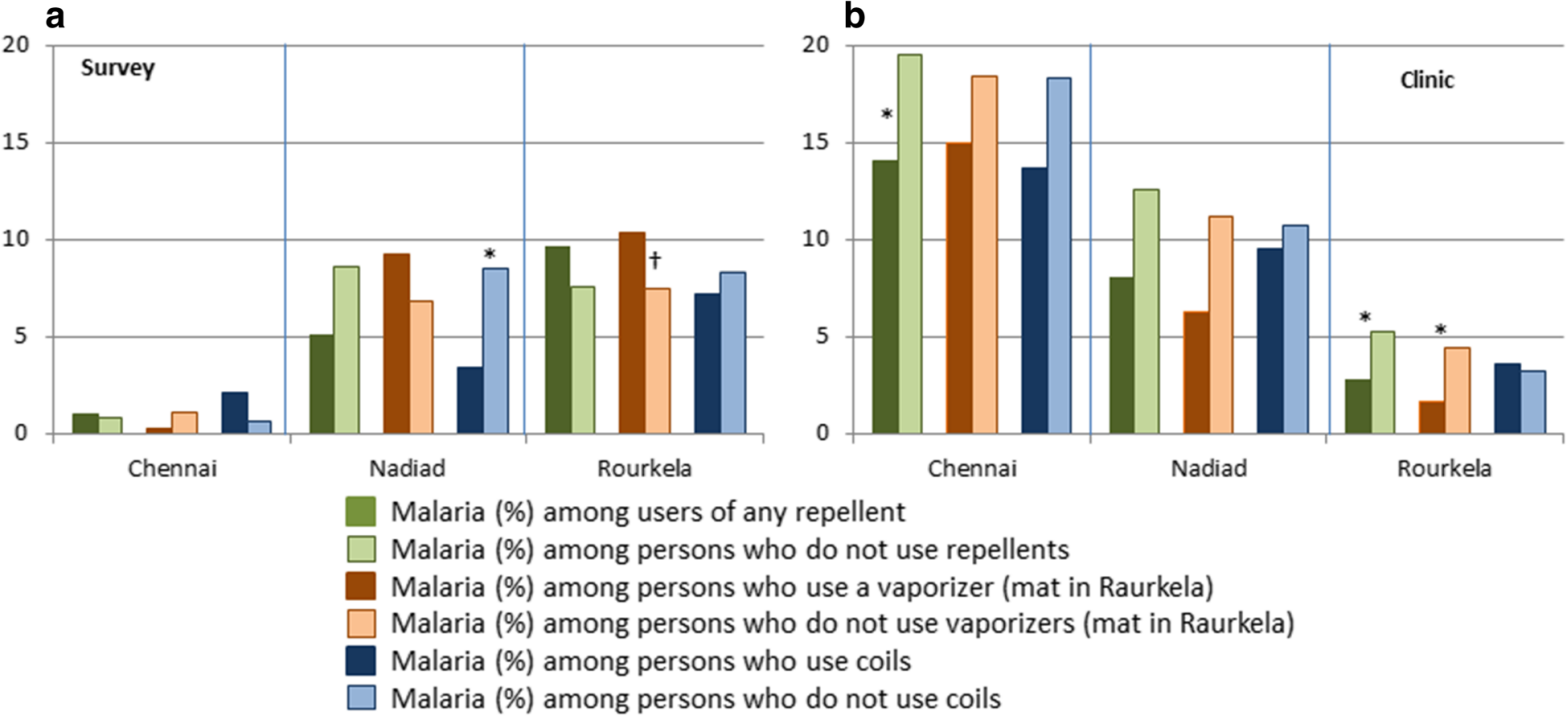
Prevalence of microscopic malaria among users and non-users of repellents by study setting and type of repellent in three sites in India, 2012–2015. The data for this graph are available in the Additional file 1: Table S7. *P < 0.05 for malaria among users *vs* no users (Chi-square test: *χ* ^*2*^ = 6.10, *df* = 1, *P* = 0.01 for coils in survey in Nadiad, *χ* ^*2*^ = 4.67, *df* = 1, *P* = 0.03 for any repellent in clinic study in Chennai, *χ* ^*2*^ = 6.25, *df* = 1, *P* = 0.01 and *χ* ^*2*^ = 10.69, *df* = 1, *P* = 0.001 for any repellent and vaporizers in clinic study in Raurkela, respectively). †In the survey in Raurkela, mats were reported to be used and not vaporizers

For the surveys and clinic studies, which included microscopy for malaria detection, association between the use of mosquito repellents and malaria was examined. Repellent use overall was associated with significantly less malaria among clinic study participants in Chennai and in Raurkela (*χ*^*2*^ = 4.67, *df* = 1, *P* = 0.03 and *χ*^*2*^ = 6.25, *df* = 1, *P* = 0.01, respectively; Fig. 3, Additional file 1: Table S7). In addition, among clinic participants the use of vaporizers was associated with significantly less malaria in Raurkela (*χ*^*2*^ = 10.69, *df* = 1, *P* = 0.001, Fig. 3). Among survey participants, the use of coils was associated with significantly less malaria only in Nadiad (*χ*^*2*^ = 6.10, *df* = 1, *P* = 0.01). Reported ITN use among survey participants in Raurkela was associated with a higher prevalence of malaria (61/469 or 13.0 % microscopic malaria among ITN users *versus* 66/1,070 or 6.2 % malaria among non-users, *χ*^*2*^ = 38.47, *df* = 1, *P* < 0.001). No association between anemia and the use of repellents was detected (data not shown).

## Discussion

We detected different patterns of repellent use in three areas in India with different types of malaria transmission. Although factors associated with repellent use differed by site and study design, they often included the SES of the household and education of the participant, in addition to season. An association between the use of repellents and malaria was detected in the clinic study in Chennai and Raurkela, and the use of coils in the survey at the Nadiad site. The different patterns of repellent use by source of population indicate that study results from clinics, a frequent source of studies in India, cannot just be extrapolated to the community.

The reported use of repellents was generally more common at the household level during the census than at the individual level in the surveys. The surveys included a more rural population in Nadiad and Raurkela, which may explain part of the difference. It is also possible that reported use at the household level was exaggerated or not applicable to every household member. The higher prevalence of repellents in Chennai and Nadiad could be due to (wealthier) urban areas being more exposed to health messages from television and newspapers and having greater access and economic ability to purchase repellents [29, 30]. Use of creams was overall low in our study for reasons that are unclear, but might include concerns about costs and safety [31]. The use of ITNs was low; India’s strategic plan for malaria only recommends ITNs and IRS in areas with an Annual Parasite Incidence of two or more, which is currently only the case at the site in Odisha and likely explains the higher use of ITNs detected in Raurkela [32].

Children and pregnant women form vulnerable groups for malaria and it is gratifying to observe that households with young children were more likely to use repellents in Chennai and Nadiad. This was not the case in the household surveys in Raurkela, the site with the highest risk of *P. falciparum* infection (Table 4). In the clinic studies in Nadiad and Raurkela, a higher use of vaporizers among children < 5 years of age was noted (Table 6). Although in Nadiad and Chennai, households with young boys appeared more likely than households with young girls to use vaporizers (Table 4), it is not clear if much value should be given to these differences, given that for girls and boys the direction and magnitude of the risk ratio was frequently similar.

**Table 6:** Factors associated with the report of personal mosquito protection among persons visiting the clinic with symptoms

	Any repellent		Vaporizer		Coil	
	Risk ratio (95 % CI)	*P*	Risk ratio (95 % CI)	*P*	Risk ratio (95 % CI)	*P*
Chennai Clinic						
At least secondary education	1.36 (1.13–1.65)	0.001	1.41 (1.09–1.82)	0.009	ns	
Occupation						
None	0.90 (0.74–1.09)	0.286	0.86 (0.65–1.13)	0.266	0.90 (0.64–1.29)	0.578
Daily wage/labor	0.58 (0.37–0.89)	0.012	0.55 (0.31–0.99)	0.045	0.43 (0.19–0.97)	0.041
Trade (self-employed)	0.87 (0.68–1.12)	0.289	0.98 (0.71–1.36)	0.903	0.70 (0.43–1.16)	0.164
Salaried	Reference		Reference		Reference	
Rainy season	ns		0.76 (0.60–0.95)	0.019	ns	
Nadiad Clinic						
Age						
< 5 years	1.29 (0.75–2.20)	0.355	2.21 (1.12–4.33)	0.021	1.02 (0.56–1.84)	0.960
5–9 years	1.61 (1.16–2.24)	0.004	1.51 (0.87–2.63)	0.140	1.52 (1.16–2.01)	0.003
10–17 years	0.99 (0.71–1.40)	0.970	1.22 (0.74–2.01)	0.438	0.68 (0.45–1.04)	0.073
> 17 years	Reference		Reference		Reference	
At least secondary education	1.33 (1.01–1.73)	0.039	2.03, 1.39–2.98	< 0.0001	ns	
Occupation						
None	0.76 (0.54–1.07)	0.112	0.68 (0.43–1.09)	0.107	ns	
Daily wage/labor	0.59 (0.39–0.90)	0.013	0.20 (0.08–0.51)	0.001		
Trade (self-employed)	0.80 (0.50–1.29)	0.363	0.73 (0.37–1.42)	0.350		
Salaried	Reference		Reference			
Rainy season	0.43 (0.31–0.61)	< 0.001	2.03 (1.39–2.98)	< 0.001	0.40 (0.26–0.63)	< 0.001
Raurkela Clinic						
Age						
< 5 years	ns		1.42 (1.16–1.73)	0.001	0.79 (0.63–0.99)	0.046
5–9 years			1.32 (1.07–1.61)	0.008	0.91 (0.76–1.11)	0.355
10–17 years			1.06 (0.88–1.26)	0.557	1.06 (0.92–1.22)	0.403
> 17 years			Reference		Reference	
At least secondary education	ns		1.34 (1.16–1.54)	< 0.001	ns	
Occupation						
None	1.01 (0.95–1.07)	0.785	1.05 (0.90–1.22)	0.561	ns	
Daily wage/labor	0.76 (0.63–0.90)	0.002	0.29 (0.15–0.55)	< 0.001		
Trade (self-employed)	1.03 (0.92–1.15)	0.602	1.08 (0.82–1.42)	0.574		
Salaried	Reference		Reference			
Malaria history past year	ns		0.72 (0.53–0.97)	0.033	ns	
Rainy season	ns		0.81 (0.72–0.90)	< 0.001	1.19 (1.07–1.32)	0.002

For tables with univariate analyses see Additional file 1: Tables S6A–C

The use of repellents is usually greater when the burden of mosquitoes increases, which is typically during or after the rainy season. However, in Nadiad at the household level the opposite pattern was detected, with repellents use occurring primarily during the dry season; it has been reported that the main mosquito burden in central Gujarat can occur after the rainy season during the dry and cooler period [33].

### Limitations

Our study has several limitations. We did not collect information on brand and costs of the repellent, and we did not verify reported use of a repellent by observation, to make sure the type of repellent was classified appropriately. The use of repellent was not quantified, e.g. we did not have information if it was used every day or occasionally or when and how many hours during the day it was used. We did not examine the use of aerosol sprays, or other types of mosquito protection that are available. We also did not check for appropriate use of ITNs.

At some sites and in some studies we detected a protective association between repellent use and malaria. In contrast, reported ITN use in Raurkela was associated with a higher risk of malaria, although as mentioned earlier, we did not confirm possession or the proper use of the nets. Possibly, the use of repellents is an indicator of people whose behavior puts them at lower risk of malaria generally. Protective effects of repellents have been reported in other observational studies in India [17]; however, a trial is the preferable design to assess efficacy. Most studies evaluating repellents conducted tests under laboratory conditions, or controlled field conditions, e.g. putting an object with a repellent in a space with mosquitoes and observing the behavior of the mosquitoes [34, 35]. Creams containing DEET have so far provided the longest protection in laboratory studies [12], though a domestically produced cream claimed similar efficacy in laboratory and field tests [36]. Studies evaluating coils did find a repellent effect [35, 37], but not a strong insecticidal (killing) effect [38]. A review of the effectiveness of coils and vapor emanators reported that coils and vaporizers induce mortality, deterrence, and repellence and reduced the ability of mosquitoes to feed on humans; however, because of the different methodologies no uniform result could be presented [10]. Guidelines for standardized laboratory tests for repellents have now been developed [39–41]. A few trials have examined the use of repellents with clinical malaria or malaria parasitemia as the outcome. A four-arm trial conducted in 2007 in an area of China with both *P. falciparum* and *P. vivax* reported a 77 % reduced prevalence of malaria (by rapid diagnostic test) among participants in households using coils, and 94 % when coils were combined with the use of insecticide treated nets (ITNs), compared to households which did not use any protection [42]. A study in Ethiopia reported a significant protective effect of daily application of a repellent cream in combination with an ITN when compared to ITNs only [43]. A trial in Thailand among 897 pregnant women after the first trimester compared a 20 % DEET cream to placebo using weekly blood smear assessments; although the women using cream had fewer episodes of parasitemia, the difference was not significant [44]. No side effects were noted in the women or their infants observed for one year [45]. A non-significant but protective effect (11.4 % reduction) was also reported from a cluster randomized trial in rural Tanzania, comparing 15 % DEET topical repellent versus placebo in a population using long lasting ITNs [46]. A plant based insect repellent showed a significant 80 % reduction in *P. vivax* in the Bolivian Amazon when used in combination with ITNs in a placebo controlled randomized trial; although the protective effect against *P. falciparum* was similar, this was not significant because of the low number of cases [47]. Soap with 20 % DEET was associated with protection against *P. falciparum* in studies from Afghanistan and Tanzania [48, 49].

### Importance for public health

Apart from ITNs, there is only limited information available about the efficacy of personal mosquito protection such as coils, mats, vaporizers or creams; fortunately a Cochrane review has been planned [50]. Repellents may form an attractive option in areas where mosquitoes bite during daytime or in the early evening, as has been reported in Asia [51, 52]. Overall, approximately two-thirds of the households and one-third of the participants in the survey and clinic studies reported the use of repellents in our studies. Taking information from other studies in India into account, the number may be even higher (Additional file 1: Table S1). Although the use of mosquito repellents may be the most prevalent preventive behavior among urban Indians in particular [29], India’s strategic plan for malaria control mentions repellents only once as an alternative method to be explored for outdoor use [32]. The consumer must rely on information provided on television and other media for recommendations for what repellent to use, and when and where, with consumer groups filling the gap [34]. The market for these types of products in India is considerable and estimated at $ 1.5 billion distributed between four main industry companies [53]. Substantial growth is anticipated because of improving levels of education in rural areas and the increasing awareness of other potentially serious mosquito-borne diseases, such as dengue, chikungunya and the newly emerging Zika virus [54]. However, it is not clear if the many and different types of products containing repellents widely available across India are actually worth the investment people make. In addition, options are limited for areas without electricity, and there are concerns about the safety of repellents and their long-term effects on health. In three villages in India where 53 % of the study participants regularly burned coils and 63 % had their doors and windows closed, high levels of small particulate matter and carbon monoxide were measured, and respiratory morbidity was higher in smaller houses where coils were used daily [55]. A laboratory study of pyrethroid concentration in indoor air during application of a coil, a vaporizer, a mat and an aerosol spray concluded that there is potential for exceeding the safety limit of some pyrethroids for children, especially among infants [56].

## Conclusions

There is considerable repellent use in India, with a wide variety of products available. Currently, their use is mainly determined by socio-economic status and level of education. Repellents can be an attractive option to reduce mosquito bites during day time, and the early evening, and are an option for the night in areas which do not qualify for ITNs or IRS. Further clinical testing of these methods and evaluation of safety will be useful for an evidence-based recommendation about how to choose and use repellents, and to consolidate their role in programs that aim to prevent vector-borne diseases.

## Abbreviations

ITN, Insecticide treated nets; IRS, Indoor residual spraying; SES, Socio-economic status

## Additional file

Additional file 1: Supplemental information. **Table S1:** Use of repellents and nets in India as reported by some studies between 2000 and 2015. **Table S2:** Comparison of characteristics of census households included and not included in the survey. **Table S3:** Use of malaria protection in three sites in India, 2012-2015. **Table S4A:** Factors associated with the report of the use of a repellent at the household level (census) in Chennai. **Table S4B:** Factors associated with the report of the use of a repellent at the household level (census) in Nadiad. **Table S4C:** Factors associated with the report of the use of a repellent at the household level (census) in Raurkela. **Table S5A:** Factors associated with the report of the use of a repellent at the individual level (survey) in Chennai. **Table S5B:** Factors associated with the report of the use of a repellent at the individual level (survey) in Nadiad. **Table S5C:** Factors associated with the report of the use of a repellent at the individual level (survey) in Raurkela. **Table S6A:** Factors associated with the report of the use of a repellent among clinic patients in Chennai. **Table S6B:** Factors associated with the report of the use of a repellent among clinic patients in Nadiad. **Table S6C:** Factors associated with the report of the use of a repellent among clinic patients in Raurkela. **Table S7:** Malaria by use of repellent, study location and type of study. (DOCX 155 kb)
