# Supplementary material for: The use of mosquito repellents at three sites in India with declining malaria transmission: surveys in the community and clinic
Source: Parasit Vectors. 2016 Jul 27;9:418. doi: 10.1186/s13071-016-1709-9 (PMC4963934; doi:10.1186/s13071-016-1709-9)
Supplement: Additional file 1: — Supplemental information. Table S1: Use of repellents and nets in India as reported by some studies between 2000 and 2015. Table S2: Comparison of characteristics of census households included and not included in the survey. Table S3: Use of malaria protection in three sites in India, 2012-2015. Table S4A: Factors associated with the report of the use of a repellent at the household level (census) in Chennai. Table S4B: Factors associated with the report of the use of a repellent at the household level (census) in Nadiad. Table S4C: Factors associated with the report of the use of a repellent at the household level (census) in Raurkela. Table S5A: Factors associated with the report of the use of a repellent at the individual level (survey) in Chennai. Table S5B: Factors associated with the report of the use of a repellent at the individual level (survey) in Nadiad. Table S5C: Factors associated with the report of the use of a repellent at the individual level (survey) in Raurkela. Table S6A: Factors associated with the report of the use of a repellent among clinic patients in Chennai. Table S6B: Factors associated with the report of the use of a repellent among clinic patients in Nadiad. Table S6C: Factors associated with the report of the use of a repellent among clinic patients in Raurkela. Table S7: Malaria by use of repellent, study location and type of study. (DOCX 155 kb) [file 13071_2016_1709_MOESM1_ESM.docx]

Additional file 1

Supplemental information to:

The use of personal malaria protection at three sites in India with declining malaria transmission; surveys in the community and clinic

Contents

[Table S1: Use of repellents and nets in India as reported by some studies between 2000 and 2015* 2](#_Toc451700575)

[Table S2: Comparison of characteristics of census households included and not included in the survey 3](#_Toc451700576)

[Table S3: Use of malaria protection in three sites in India, 2012-2015 4](#_Toc451700577)

[Table S4A: Factors associated with the report of the use of a repellent at the household level (census) in Chennai 5](#_Toc451700578)

[Table S4B: Factors associated with the report of the use of a repellent at the household level (census) in Nadiad 6](#_Toc451700579)

[Table S4C: Factors associated with the report of the use of a repellent at the household level (census) in Raurkela 8](#_Toc451700580)

[Table S5A: Factors associated with the report of the use of a repellent at the individual level (survey) in Chennai 10](#_Toc451700581)

[Table S5B: Factors associated with the report of the use of a repellent at the individual level (survey) in Nadiad 12](#_Toc451700582)

[Table S5C: Factors associated with the report of the use of a repellent at the individual level (survey) in Raurkela 14](#_Toc451700583)

[Table S6A: Factors associated with the report of the use of a repellent among clinic patients in Chennai 16](#_Toc451700584)

[Table S6B: Factors associated with the report of the use of a repellent among clinic patients in Nadiad 18](#_Toc451700585)

[Table S6C: Factors associated with the report of the use of a repellent among clinic patients in Raurkela 20](#_Toc451700586)

[Table S7: Malaria by use of repellent, study location and type of study 22](#_Toc451700587)

[References 23](#_Toc451700588)

Table S1: Use of repellents and nets in India as reported by some studies between 2000 and 2015*

| **Author** | **Location, year** | **Design, Sample size** | **Urban** | **Rural** |
| --- | --- | --- | --- | --- |
| Snehalatha 2003 [1] | Pondicherry, before 2003 | Survey, households  Urban: N=300  Rural: N=100 | Any method: 99.3%  Coils: 38-58%  Mats: 36-49% | Any method: 73.0%  Coils: 21-23%  Electric fans: 43-55% |
| Babu 2007 [2] | Orissa, before 2007 | Survey, persons  Urban: N=300. Rural: N=300 | Any method: 92.3%  Coil: 79.3%  Mat: 11.7%  Liquid vaporizer: 21.0%  Spray: 0.3%  Smoke: 9.7%  Bednet: 75.7% | Any method: 64.3%  Coil: 61.7%  Mat: 0.7%  Liquid vaporizer: 5.3%  Spray: 0%  Smoke: 8.3%  Bednet: 57.7% |
| Singh 2013 [3] | Bihar and Jharkhand,  2008 and 2010, respectively | Survey, households  Rural: N=426 |  | Any repellent: 73.2%  Coils: 12.4%  Cow dung and neem leaves: 16.2%  ITNs: 67.8%  IRS: 89.7% |
| Kowli 2010 [4]* | Mumbai, Maharashtra, 2008 | Survey, households Urban: N=200 | Creams: 32.5%  Coils: 44.5%  Mats/vaporizers: 42.0% |  |
| Dhawan 2014 [5] | Mumbai, Maharashtra, 2010-2011 | Survey: households  Urban: N=89 Rural: N=25 | Coils: city 20%, construction site 50%, slum 27%  Liquid repellents: city 67%, construction site 10%, slums 33%  Spray: city 33%, construction site 3%, slum 7%  Net: city 0%, construction site, slum 7%  ITN: city and construction site 0%, slum 13% | Coils: 55%  Liquid repellents: 17%  Spray:14%  Net: 14%  ITN: 3% |
| Vala 2013 [6] | Rajkot, Gujarat, 2011 | Survey, households  Urban: N=216  Rural: N=216 | Any method: 96.3%  Liquid vaporizer: 75.5%  Coils and mats: 11.6%  Cream: 0  Screening window: 14.8%  Net: 9.3% | Any method: 84.7%  Liquid vaporizer: 29.2%  Coils and mats: 15.3%  Cream: 3%  Screening window: 2.3%  Net: 21.8% |
| Chitra 2013 [7] | Tamil Nadu, 2010 | Survey, households  Rural: N=143 |  | Any method: 94.4%  Coils: 75%  Vaporizers: 36%  Nets: 5% |
| Bhattacharyya 2015 [8] | Meghalaya, 2013 | Survey, households  Urban: N=200 | Any method: 59%  Coils: 42%  Nets: 13%  ITNs: 3%  “Burnt cloths”: 4% |  |

*Where available, net and ITN use reported for comparison

†Protective effect repellents: history of malaria among 5/486 users and 29/485 non-users

Table S2: Comparison of characteristics of census households included and not included in the survey

|  | **Chennai** | | **Nadiad** | | **Raurkela** | |
| --- | --- | --- | --- | --- | --- | --- |
|  | **Census HH not in survey (N=1077)** | **Census HH in survey (N=406)** | **Census HH not in survey (N=1393)** | **Census HH in survey (N=439)** | **Census HH not in survey (N=786)** | **Census HH in survey (N=418)** |
| Electricity (%) | 1074 (99.7) | 405 (99.8) | 1363 (97.9) | 401 (91.3)* | 693 (88.2) | 282 (67.5)* |
| Livestock on site | 18/1060 (1.7) | 10/404 (2.5) | 94 (6.8) | 123 (28.0)* | 344 (43.8) | 355 (84.9)* |
| Average number of people in the household | 4.1, sd 1.6  Range 1-21 | 4.5, sd 1.3*  Range 2-12 | 5.4, sd 2.5  Range 1-16 | 4.6, sd 2.5*  Range 1-12 | 5.1, sd 2.2  Range 1-17 | 5.4, sd 2.0*  Range 2-24 |
| Young child in household† | 254 (23.6) | 120 (29.6)* | 360/1393 (25.8) | 103/439 (23.5) | 312 (39.7) | 182 (43.5) |
| Young male child | 133 (12.4) | 82 (20.2)* | 212/1393 (15.2) | 65/439 (14.8) | 193 (24.6) | 112 (26.8) |
| Young female child | 150 (13.9) | 56 (13.8) | 200/1393 (14.4) | 55/439 (12.5) | 185 (23.5) | 102 (24.4) |
| Male head of household | 914/1056 (86.6) | 356/401 (88.8) | 1150/1337 (86.0) | 354/427 (82.9) | 663 (84.4) | 388 (92.8)* |
| Education head of household secondary plus | 428/882 (48.5) | 125/406 (37.3) | 373/1337 (27.9) | 68/427 (15.9)* | 40 (5.1) | 13 (3.1) |
| Head of household salaried employment | 404/1038 (38.9) | 139/395 (35.2) | 297/1337 (22.2) | 38/427 (8.9)* | 103/784 (13.1) | 17 (4.1)* |
| ≥ 1 Person with at least secondary education in hh | 709 (65.8) | 262 (64.5) | 788 (56.6) | 157 (35.8)* | 246 (31.3) | 55 (13.2)* |
| SES | N=1011 | N=389 | N=1337 | N=427 | N=782 | N=418 |
| Rich | 228 (22.6) | 59 (15.2)* | 340 (25.4) | 38 (8.9)* | 234 (29.9) | 9 (2.2)* |
| 4 | 199 (19.7) | 80 (20.6) | 298 (22.3) | 43 (10.1) | 225 (28.8) | 15 (3.6) |
| 3 | 204 (20.2) | 83 (21.3) | 300 (22.4) | 44 (10.3) | 135 (17.3) | 103 (24.6) |
| 2 | 193 (19.1) | 91 (23.4) | 247 (18.5) | 103 (24.1) | 130 (16.6) | 190 (45.5) |
| Poor | 187 (18.5) | 76 (19.5) | 152 (11.4) | 199 (46.6) | 58 (7.4) | 101 (24.2) |
| Head of household had malaria in previous year | 69/1058 (6.5) | 25/402 (6.2) | 45/1337 (3.4) | 10/427 (2.3) | 51/784 (6.5) | 81 (19.4)* |
| ≥ 1 person with malaria in the previous year | 160 (14.9) | 77 (19.0) | 124 (8.9) | 22 (5.0)* | 111 (14.1) | 149 (35.7)* |
| 1 person | 124 (11.5) | 63 (15.5) | 104 (7.5) | 17 (3.9) | 62 (7.9) | 77 (18.8)* |
| 2 persons | 21 (2.0) | 12 (3.0) | 15 (1.1) | 3 (0.7) | 33 (4.2) | 49 (11.7) |
| > 2 persons | 15 (1.4) | 2 (0.5) | 5 (0.4) | 2 (0.5) | 16 (2.0) | 23 (5.5) |
| Enrolment in rainy season | 341 (31.7) | 121 (29.8) | 2 (0.1) | 107 (24.4)* | 90 (11.5) | 92 (22.0)* |

*P<0.05 comparing household included and not included in the survey using the chi-square test or *T*-test

†young child: < 5 years of age

Note: 20 households were excluded because of lack of information on repellents: 12 in Chennai, 6 in Nadiad, and 8 in Raurkela

Table S3: Use of malaria protection in three sites in India, 2012-2015

|  | **Census: reported for households** | | | **Survey: reported by participants** | | | **Clinic: reported by participants** | | |
| --- | --- | --- | --- | --- | --- | --- | --- | --- | --- |
| Personal malaria protection | Chennai N=1483 | Nadiad N=1832 | Raurkela N=1204 | Chennai  N=928 | Nadiad  N=796 | Raurkela  N=1539 | Chennai  N=1054 | Nadiad  N=685 | Raurkela  N=1875 |
| Use of any repellent | 881 (59.4)§ | 1411 (77.0)† | 680 (56.5) | 422 (45.5)* | 317 (39.8)† | 487 (31.6) | 355 (33.7)* | 344 (50.2)† | 1461 (77.9) |
| Coil | 236 (15.9)* | 767 (41.9)† | 404 (33.6) | 140 (15.1)* | 208 (26.1)† | 83 (5.4) | 139 (13.2)* | 190 (27.7)† | 798 (42.6) |
| Vaporizers | 655 (44.2)‡ | 769 (42.0)† | 73 (6.1) | 310 (33.4)* | 109 (13.7)† | 9 (0.6) | 227 (21.5)* | 112 (16.4)† | 727 (38.8) |
| Cream | 26 (1.8)‡ | 35 (1.9)† | 3 (0.3) | 26 (2.8)* | 1 (0.1) | 0 (0.0) | 11 (1.0)‡ | 3 (0.4) | 4 (0.2) |
| Mat | 36 (2.4)* | 126 (6.9)† | 244 (20.3) | 1 (0.1)* | 22 (2.8)† | 396 (25.7) | 8 (0.8)* | 56 (8.2)† | 78 (4.2) |
| Use of net | 40/1473 (2.7)* | 449 (24.5)† | 928 (77.1) | 48 (5.2)* | 106 (13.3)† | 1314 (85.4) | 53 (5.0)* | 79 (11.5)† | 816 (43.5) |
| Use of ITN | 1/1473 (0.1)* | 23 (1.3)† | 394 (32.8) | 0 (0.0)‡ | 5 (0.6)† | 469 (30.5) | 0 (0.0) | 0 (0.0) | 1 (0.05) |

Note: data is presented in Figure 1 in the manuscript. ITN: insecticide treated net

**P*<0.05 comparing Chennai to Nadiad and Raurkela (Chi-square test)

†*P*<0.05 comparing Nadiad to Raurkela (Chi-square test)

‡*P*<0.05 comparing Chennai to Raurkela (Chi-square test)

§*P*<0.05 comparing Chennai to Nadiad (Chi-square test)

Table S4A: Factors associated with the report of the use of a repellent at the household level (census) in Chennai

|  |  | |  | | **Multivariate** | |
| --- | --- | --- | --- | --- | --- | --- |
| **Chennai: repellents** | **Users in HH with factor** | **Users in HH without factor** | **RR, 95% CI** | ***P*** | **RR, 95% CI** | ***P*** |
| **Factor** |  |  |  |  |  |  |
| Young female child | 125/206 (60.7) | 756/1277 (59.2) | 1.02, 0.91-1.15 | 0.686 | 1.05, 0.93-1.18 | 0.455 |
| Young male child | 143/215 (66.5) | 738/1268 (58.2) | 1.14, 1.03-1.27 | 0.013 | 1.17, 1.05-1.29 | 0.003 |
| Male head of HH | 753/1270 (59.3) | 109/187 (58.3) | 1.02, 0.89-1.16 | 0.796 | NS |  |
| Member with secondary education | 641/971 (66.0) | 240/512 (46.9) | 1.41, 1.27-1.56 | <0.001 | 1.26, 1.12-1.41 | <0.001 |
| Socio-economic status |  |  |  |  |  |  |
| High | 187/287 (65.2) |  | 1.34, 1.15-1.56 | <0.001 | 1.37, 1.18-1.59 | <0.001 |
| 2 | 186/279 (66.7) |  | 1.37, 1.18-1.59 | <0.001 | 1.32, 1.14-1.53 | <0.001 |
| 3 | 157/287 (54.7) |  | 1.12, 0.96-1.32 | 0.159 | 1.06, 0.90-1.24 | 0.487 |
| 4 | 165/284 (58.1) |  | 1.19, 1.02-1.40 | 0.029 | 1.13, 0.97-1.32 | 0.110 |
| Low | 128/263 (48.7) |  | Reference |  | Reference |  |
| Person with episode of malaria in previous year | 137/237 (57.8) | 744/1246 (59.7) | 0.97, 0.86-1.09 | 0.590 | NS |  |
| Rainy season at interview | 334/462 (72.3) | 547/1021 (53.6) | 1.35, 1.25-1.46 | <0.001 | 1.33, 1.21-1.46 | <0.001 |
| **Chennai: vaporizers** | **Users in HH with factor** | **Users in HH without factor** | **RR, 95% CI** | ***P*** | **RR, 95% CI** | ***P*** |
| **Factor** |  |  |  |  |  |  |
| Young female child | 100/206 (48.5) | 555/1277 (43.7) | 1.12, 0.96-1.30 | 0.159 | 1.12, 0.96-1.30 | 0.138 |
| Young male child | 109/215 (50.7) | 546/1268 (43.1) | 1.18, 1.02-1.36 | 0.029 | 1.22, 1.06-1.40 | 0.006 |
| Male head of HH | 580/1270 (45.7) | 61/187 (32.6) | 1.40, 1.13-1.74 | 0.002 | 1.38, 1.18-1.60 | <0.001 |
| Person with secondary education in HH | 487/971 (50.2) | 168/512 (32.8) | 1.53, 1.33-1.76 | <0.001 | 1.27, 1.02-1.58 | 0.035 |
| Socio-economic status |  |  |  |  |  |  |
| High | 163/287 (56.8) |  | 2.33, 1.84-2.95 | <0.001 | 2.22, 1.75-2.81 | <0.001 |
| 2 | 159/279 (57.0) |  | 2.34, 1.85-2.97 | <0.001 | 2.17, 1.70-2.73 | <0.001 |
| 3 | 112/287 (39.0) |  | 1.60, 1.24-2.07 | <0.001 | 1.46, 1.13-1.89 | 0.004 |
| 4 | 113/284 (39.8) |  | 1.64, 1.26-2.11 | <0.001 | 1.55, 1.20-2.00 | 0.001 |
| Low | 64/263 (24.3) |  | Reference |  |  |  |
| Person with episode of malaria in previous year | 97/237 (40.9) | 558/1246 (44.8) | 0.91, 0.77-1.08 | 0.285 | NS |  |
| Rainy season at interview | 225/462 (48.7) | 430/1021 (42.1) | 1.16, 1.03-1.30 | 0.016 | 1.14, 1.01-1.30 | 0.038 |
| **Chennai: coils** | **Users in HH with factor** | **Users in HH without factor** | **RR, 95% CI** | ***P*** | **RR, 95% CI** | ***P*** |
| **Factor** |  |  |  |  |  |  |
| Young female child | 29/206 (14.1) | 207/1277 (16.2) | 0.87, 0.61-1.24 | 0.442 | NS |  |
| Young male child | 36/215 (16.7) | 200/1268 (15.8) | 1.06, 0.77-1.47 | 0.718 | NS |  |
| Male head of HH | 183/1270 (14.4) | 49/187 (26.2) | 0.55, 0.42-0.72 | <0.001 | 0.63, 0.48-0.82 | 0.001 |
| Person with secondary education in HH | 168/971 (17.3) | 68/512 (13.3) | 1.30, 1.00-1.69 | 0.047 | NS |  |
| Socio-economic status |  |  |  |  |  |  |
| High | 17/287 (5.9) |  | 0.22, 0.13-0.36 | <0.001 | 0.28, 0.17-0.47 | <0.001 |
| 2 | 36/279 (12.9) |  | 0.48, 0.33-0.69 | <0.001 | 0.52, 0.36-0.74 | <0.001 |
| 3 | 42/287 (14.6) |  | 0.54, 0.38-0.76 | <0.001 | 0.54, 0.39-0.76 | <0.001 |
| 4 | 59/284 (20.8) |  | 0.77, 0.57-1.04 | 0.089 | 0.73, 0.54-0.98 | 0.037 |
| Low | 71/263 (27.0) |  | Reference |  | Reference |  |
| Person with episode of malaria in previous year | 43/237 (18.1) | 193/1246 (15.5) | 1.17, 0.87-1.58 | 0.301 | NS |  |
| Rainy season at interview | 124/462 (26.8) | 112/1021 (11.0) | 2.45, 1.94-3.08 | <0.001 | 2.13, 1.68-2.70 | <0.001 |

Abbreviations: CI: confidence interval, HH: household, NS: not significant, RR: risk ratio (prevalence ratio)

Table S4B: Factors associated with the report of the use of a repellent at the household level (census) in Nadiad

|  |  |  | **Univariate analysis** | | **Multivariate analysis** | |
| --- | --- | --- | --- | --- | --- | --- |
| **Nadiad: repellents** | **Users in HH with factor** | **Users in HH without factor** | **RR, 95% CI** | ***P*** | **RR, 95% CI** | ***P*** |
| **Factor** |  |  |  |  |  |  |
| Young female child | 214/255 (83.9) | 1197/1577 (75.9) | 1.11, 1.04-1.17 | 0.001 | 1.12, 1.05-1.19 | <0.001 |
| Young male child | 227/277 (82.0) | 1184/1555 (76.1) | 1.08, 1.01-1.14 | 0.020 | 1.09, 1.03-1.15 | 0.005 |
| Male head of HH | 1174/1504 (78.1) | 185/260 (71.2) | 1.10, 1.01-1.19 | 0.027 | NS |  |
| Member with secondary education | 814/945 (86.1) | 597/887 (67.3) | 1.28, 1.21-1.35 | <0.001 | NS |  |
| Socio-economic status |  |  |  |  |  |  |
| High | 352/378 (93.1) |  | 2.07, 1.84-2.33 | <0.001 | 1.93, 1.72-2.17 | <0.001 |
| 2 | 316/341 (92.7) |  | 2.06, 1.83-2.32 | <0.001 | 1.93, 1.72-2.17 | <0.001 |
| 3 | 299/344 (86.9) |  | 1.93, 1.71-2.18 | <0.001 | 1.82, 1.62-2.05 | <0.001 |
| 4 | 234/350 (66.9) |  | 1.49, 1.29-1.70 | <0.001 | 1.43, 1.26-1.63 | <0.001 |
| Low | 158/351 (45.0) |  | Reference |  | Reference |  |
| Person with episode of malaria in previous year | 113/146 (77.4) | 1298/1686 (77.0) | 1.01, 0.92-1.10 | 0.909 | NS |  |
| Rainy season at interview | 32/109 (29.4) | 1379/1723 (80.0) | 0.37, 0.27-0.49 | <0.001 | 0.46, 0.34-0.61 | <0.001 |
| **Nadiad: vaporizers** | **Users in HH with factor** | **Users in HH without factor** | **RR, 95% CI** | ***P*** | **RR, 95% CI** | ***P*** |
| **Factor** |  |  |  |  |  |  |
| Young female child | 92/255 (36.1) | 677/1577 (42.9) | 0.84, 0.71-0.99 | 0.049 | 0.95, 0.82-1.10 | 0.486 |
| Young male child | 115/277 (41.5) | 654/1555 (42.1) | 0.99, 0.85-1.15 | 0.867 | 1.14, 1.00-1.30 | 0.045 |
| Male head of HH | 653/1504 (43.4) | 84/260 (32.3) | 1.34, 1.12-1.62 | 0.002 | NS |  |
| Person with secondary education in HH | 582/945 (61.6) | 187/887 (21.1) | 2.92, 2.55-3.35 | <0.001 | 1.48, 1.29-1.70 | <0.001 |
| Socio-economic status |  |  |  |  |  |  |
| High | 283/378 (74.9) |  | 23.89 (13.32-42.86) | <0.001 | 16.63, 9.17-30.14 | <0.001 |
| 2 | 218/341 (63.9) |  | 20.40, 11.34-36.69 | <0.001 | 15.25, 8.41-27.62 | <0.001 |
| 3 | 161/344 (46.8) |  | 14.93, 8.26-27.01 | <0.001 | 12.18, 6.71-22.12 | <0.001 |
| 4 | 64/350 (18.3) |  | 5.83, 3.13-10.87 | <0.001 | 5.37, 2.90-9.98 | <0.001 |
| Low | 11/351 (3.1) |  | Reference |  | Reference |  |
| Person with episode of malaria in previous year | 53/146 (36.3) | 716/1686 (42.5) | 0.85, 0.68-1.07 | 0.166 | NS |  |
| Rainy season at interview | 8/109 (7.3) | 761/1723 (44.2) | 0.17, 0.09-0.32 | <0.001 | 0.36, 0.18-0.70 | 0.003 |
| **Nadiad: coils** | **Users in HH with factor** | **Users in HH without factor** | **RR, 95% CI** | ***P*** | **RR, 95% CI** | ***P*** |
| **Factor** |  |  |  |  |  |  |
| Young female child | 138/255 (54.1) | 629/1577 (39.9) | 1.36, 1.19-1.54 | <0.001 | 1.24, 1.09-1.41 | 0.001 |
| Young male child | 143/277 (51.6) | 624/1555 (40.1) | 1.29, 1.13-1.46 | <0.001 | 1.18, 1.04-1.34 | 0.010 |
| Male head of HH | 642/1504 (42.7) | 103/260 (39.6) | 1.08, 0.92-1.27 | 0.364 | NS |  |
| Person with secondary education in HH | 339/945 (35.9) | 428/887 (48.3) | 0.74, 0.67-0.83 | <0.001 | 0.81, 0.72-0.92 | 0.001 |
| Socio-economic status |  |  |  |  |  |  |
| High | 122/378 (32.3) |  | 0.80, 0.66-0.98 | 0.027 | 0.88, 0.71-1.08 | 0.223 |
| 2 | 136/341 (39.9) |  | 0.99, 0.83-1.19 | 0.938 | 1.04, 0.86-1.26 | 0.679 |
| 3 | 164/344 (47.7) |  | 1.19, 1.00-1.41 | 0.047 | 1.19, 1.01-1.40 | 0.041 |
| 4 | 182/350 (52.0) |  | 1.29, 1.10-1.52 | 0.002 | 1.26, 1.08-1.47 | 0.004 |
| Low | 141/351 (40.2) |  | Reference |  | Reference |  |
| Person with episode of malaria in previous year | 77/146 (52.7) | 690/1686 (40.9) | 1.29, 1.09-1.52 | 0.002 | NS |  |
| Rainy season at interview | 22/109 (20.2) | 745/1723 (43.2) | 0.47, 0.32-0.68 | <0.001 | 0.44, 0.37-0.48 | <0.001 |

Abbreviations: CI: confidence interval, HH: household, NS: not significant, RR: risk ratio (prevalence ratio)

Table S4C: Factors associated with the report of the use of a repellent at the household level (census) in Raurkela

|  |  |  | **Univariate analysis** | | **Multivariate analysis** | |
| --- | --- | --- | --- | --- | --- | --- |
| **Raurkela: repellents** | **Users in HH with factor** | **Users in HH without factor** | **RR, 95% CI** | ***P*** | **RR, 95% CI** | ***P*** |
| **Factor** |  |  |  |  |  |  |
| Young female child | 160/287 (55.8) | 520/917 (56.7) | 0.98, 0.87-1.11 | 0.776 | NS |  |
| Young male child | 156/305 (51.2) | 524/899 (58.3) | 0.88, 0.78-0.99 | 0.037 | NS |  |
| Male head of HH | 572/1051 (54.4) | 108/153 (70.6) | 0.77, 0.69-0.87 | <0.001 | 0.87, 0.78-0.97 | 0.013 |
| Member with secondary education | 212/301 (70.4) | 468/903 (51.8) | 1.36, 1.23-1.50 | <0.001 | NS |  |
| Socio-economic status |  |  |  |  |  |  |
| High | 206/243 (84.8) |  | 1.59, 1.36-1.85 | <0.001 | 1.89, 1.59-2.24 | <0.001 |
| 2 | 188/240 (78.3) |  | 1.47, 1.25-1.72 | <0.001 | 1.67, 1.41-1.97 | <0.001 |
| 3 | 74/238 (31.1) |  | 0.58, 0.46-0.74 | <0.001 | 0.65, 0.52-0.81 | <0.001 |
| 4 | 123/320 (38.4) |  | 0.72, 0.59-0.88 | 0.001 | 0.74, 0.62-0.88 | 0.001 |
| Low | 85/159 (53.5) |  | Reference |  | Reference |  |
| Person with episode of malaria in previous year | 77/260 (29.6) | 603/944 (63.9) | 0.46, 0.38-0.56 | <0.001 | 0.63, 0.53-0.75 | <0.001 |
| Rainy season at interview | 77/182 (42.3) | 603/1022 (59.0) | 0.72, 0.60-0.86 | <0.001 | NS |  |
| ITN use | 236/394 (59.9) | 444/809 (54.9) | 1.09, 0.99-1.21 | 0.093 | 1.66, 1.45-1.90 | <0.001 |
| **Raurkela: mats** | **Mat users in HH with factor** | **Mat users in HH without factor** | **RR, 95% CI** | ***P*** | **RR, 95% CI** | ***P*** |
| **Factor** |  |  |  |  |  |  |
| Young female child | 56/287 (19.5) | 188/917 (20.5) | 0.95, 0.73-1.24 | 0.717 | NS |  |
| Young male child | 65/305 (21.3) | 179/899 (19.9) | 1.07, 0.83-1.38 | 0.598 | NS |  |
| Male head of HH | 224/1051 (21.3) | 20/155 (13.1) | 1.63, 1.07-2.49 | 0.024 | 0.69, 0.50-0.95 | 0.022 |
| Person with secondary education in HH | 19/301 (6.3) | 225/903 (24.9) | 0.25, 0.16-0.40 | <0.001 | NS |  |
| Socio-economic status |  |  |  |  |  |  |
| High | 1/243 (0.4) |  | 0.01, 0.00-0.06 | <0.001 | 0.02, 0.00-0.12 | <0.001 |
| 2 | 7/240 (2.9) |  | 0.06, 0.03-0.12 | <0.001 | 010, 0.05-0.22 | <0.001 |
| 3 | 41/238 (17.2) |  | 0.34, 0.25-0.46 | <0.001 | 0.41, 0.31-0.55 | <0.001 |
| 4 | 114/320 (35.6) |  | 0.70, 0.57-0.86 | 0.001 | 0.69, 0.57-0.83 | <0.001 |
| Low | 81/159 (50.9) |  | Reference |  | Reference |  |
| Person with episode of malaria in previous year | 47/260 (18.1) | 197/944 (20.9) | 0.87, 0.65-1.15 | 0.327 |  |  |
| Rainy season at interview | 61/182 (33.5) | 183/1022 (17.9) | 1.87, 1.47-2.39 | <0.001 |  |  |
| ITN use | 194/394 (49.2) | 50/809 (6.2) | 7.97, 5.98-10.61 | <0.001 | 4.20, 3.15-5.59 | <0.001 |
| **Raurkela: coils** | **Coil users in HH with factor** | **Coil users in HH without factor** | **RR, 95% CI** | ***P*** | **RR, 95% CI** | ***P*** |
| **Factor** |  |  |  |  |  |  |
| Young female child | 96/287 (33.5) | 308/917 (33.6) | 1.00, 0.83-1.20 | 0.965 | NS |  |
| Young male child | 85/305 (27.9) | 319/899 (35.5) | 0.79, 0.64-0.96 | 0.018 | NS |  |
| Male head of HH | 319/1051 (30.4) | 85/153 (55.6) | 0.55, 0.46-0.65 | <0.001 | 0.88, 0.79-0.99 | 0.037 |
| Person with secondary education in HH | 174/301 (57.8) | 230/903 (25.5) | 2.27, 1.96-2.63 | <0.001 | NS |  |
| Socio-economic status |  |  |  |  |  |  |
| High | 187/243 (77.0) |  | 61.18, 15.41-242.9 | <0.001 | 47.96, 12.12-189.71 | <0.001 |
| 2 | 170/240 (70.8) |  | 56.31, 14.17-223.7 | <0.001 | 45.21, 11.41-179.21 | <0.001 |
| 3 | 33/238 (13.9) |  | 11.02, 2.68-45.29 | 0.001 | 11.36, 2.75-46.95 | 0.001 |
| 4 | 8/320 (2.5) |  | 1.99, 0.43-9.25 | 0.381 | 2.24, 0.47-10.57 | 0.309 |
| Low | 2/159 (1.3) |  | Reference |  | Reference |  |
| Person with episode of malaria in previous year | 29/260 (11.2) | 375/944 (39.7) | 0.28, 0.20-0.40 | <0.001 | 0.68, 0.52-0.90 | 0.007 |
| Rainy season at interview | 16/182 (8.8) | 388/1022 (38.0) | 0.23, 0.14-0.37 | <0.001 | 0.54, 0.35-0.83 | 0.005 |
| Use of ITNs | 39/394 (9.9) | 365/809 (45.1) | 0.22, 0.16-0.30 | <0.001 | 0.71, 0.55-0.92 | 0.010 |
| **Raurkela: ITNs** | **Users in HH with factor** | **Users in HH without factor** | **RR, 95% CI** | ***P*** | **RR, 95% CI** | ***P*** |
| **Factor** |  |  |  |  |  |  |
| Young female child | 103/287 (35.9) | 291/916 (31.8) | 1.13, 0.94-1.35 | 0.188 | NS |  |
| Young male child | 100/305 (32.8) | 294/898 (32.7) | 1.00, 0.83-1.21 | 0.988 | NS |  |
| Male head of HH | 367/1051 (34.9) | 27/152 (17.8) | 1.97, 1.38-2.79 | <0.001 | NS |  |
| Person with secondary education in HH | 57/301 (18.9) | 337/902 (37.4) | 0.51, 0.40-0.65 | <0.001 | NS |  |
| Socio-economic status |  |  |  |  |  |  |
| High | 15/243 (6.2) |  | 0.11, 0.07-0.19 | <0.001 | 0.19, 0.12-0.33 | <0.001 |
| 2 | 32/240 (13.3) |  | 0.25, 0.17-0.35 | <0.001 | 0.40, 0.28-0.57 | <0.001 |
| 3 | 87/238 (36.6) |  | 0.68, 0.54-0.84 | <0.001 | 0.86, 0.70-1.06 | 0.149 |
| 4 | 174/319 (54.6) |  | 1.01, 0.85-1.20 | 0.925 | 1.05, 0.89-1.23 | 0.561 |
| Low | 86/159 (54.1) |  | Reference |  | Reference |  |
| Person with episode of malaria in previous year | 94/260 (36.2) | 300/943 (31.8) | 1.14, 0.94-1.37 | 0.179 | NS |  |
| Rainy season at interview | 106/183 (58.2) | 288/1021 (28.2) | 2.06, 1.76-2.42 | <0.001 | 1.41, 1.22-1.63 | <0.001 |
| Use of mats | 194/244 (79.5) | 200/959 (20.9) | 3.81, 3.32-4.38 | <0.001 | 2.38, 2.05-2.75 | <0.001 |
| Use of coils | 39/404 (9.7) | 355/799 (44.4) | 0.22, 0.16-0.30 | <0.001 | NS |  |

Abbreviations: CI: confidence interval, HH: household, NS: not significant, RR: risk ratio (prevalence ratio)

Table S5A: Factors associated with the report of the use of a repellent at the individual level (survey) in Chennai

|  |  | | **Univariate analysis** | | **Multivariate analysis*** | |
| --- | --- | --- | --- | --- | --- | --- |
| **Chennai: repellents** | **User with factor** | **User without factor** | **RR, 95% CI** | ***P*** | **RR, 95% CI** | ***P*** |
| **Factor** |  |  |  |  |  |  |
| Male | 166/359 (46.2) | 256/659 (45.0) | 1.03, 0.89-1.19 | 0.709 |  |  |
| Age |  |  |  |  |  |  |
| <5 years | 11/22 (50.0) |  | 1.09, 0.71-1.66 | 0.698 |  |  |
| 5-9 years | 18/48 (37.5) |  | 0.82, 0.56-1.18 | 0.285 |  |  |
| 10-17 years | 34/76 (44.7) |  | 0.97, 0.75-1.26 | 0.839 |  |  |
| >17 years | 359/781 (46.0) |  | Reference |  |  |  |
| Person with secondary education | 267/533 (50.1) | 155/395 (39.2) | 1.27, 1.10-1.48 | 0.001 | 1.28, 1.09-1.50 | 0.002 |
| Socio-economic status |  |  |  |  |  |  |
| High | 42/109 (38.5) |  | 0.87, 0.65-1.17 | 0.353 |  |  |
| 2 | 89/187 (47.6) |  | 1.07, 0.85-1.35 | 0.543 |  |  |
| 3 | 79/167 (47.3) |  | 1.07, 0.84-1.35 | 0.588 |  |  |
| 4 | 99/191 (51.8) |  | 1.17, 0.94-1.46 | 0.166 |  |  |
| Low | 70/158 (44.3) |  | Reference |  |  |  |
| Person with episode of malaria in previous year | 19/53 (35.9) | 403/875 (46.1) | 0.78, 0.54-1.12 | 0.181 |  |  |
| Rainy season at interview | 242/531 (45.6) | 180/397 (45.3) | 1.01, 0.87-1.16 | 0.943 |  |  |
| **Chennai: vaporizers** | **Users with factor** | **Users without factor** | **RR, 95% CI** | ***P*** | **RR, 95% CI** | ***P*** |
| **Factor** |  |  |  |  |  |  |
| Male | 120/359 (33.4) | 190/569 (33.4) | 1.00, 0.83-1.21 | 0.991 |  |  |
| Age |  |  |  |  |  |  |
| <5 years | 10/22 (45.5) |  | 1.34, 0.84-2.15 | 0.215 | NS |  |
| 5-9 years | 11/48 (22.9) |  | 0.68, 0.40-1.15 | 0.149 |  |  |
| 10-17 years | 25/76 (32.9) |  | 0.97, 0.70-1.36 | 0.874 |  |  |
| >17 years | 264/781 (33.8) |  | Reference |  |  |  |
| Person with secondary education | 199/533 (37.3) | 111/395 (28.1) | 1.32, 1.10-1.61 | 0.004 | NS |  |
| Socio-economic status |  |  |  |  |  |  |
| High | 38/109 (34.9) |  | 1.62, 1.09-2.40 | 0.016 | 1.62, 0.90-2.91 | 0.106 |
| 2 | 72/187 (38.5) |  | 1.79, 1.26-2.54 | 0.001 | 1.79, 1.04-3.09 | 0.036 |
| 3 | 65/167 (38.9) |  | 1.81, 1.27-2.58 | 0.001 | 1.81, 1.05-3.13 | 0.034 |
| 4 | 72/191 (37.7) |  | 1.75, 1.24-2.48 | 0.002 | 1.75, 1.02-3.02 | 0.043 |
| Low | 34/158 (21.5) |  | Reference |  | Reference |  |
| Person with episode of malaria in previous year | 15/53 (28.3) | 295/875 (33.7) | 0.84, 0.54-1.30 | 0.434 |  |  |
| Rainy season at interview | 194/531 (36.5) | 116/397 (29.2) | 1.25, 1.03-1.51 | 0.021 | NS |  |
| **Chennai: coils** | **Users with factor** | **Users without factor** | **RR, 95% CI** | ***P*** | **RR, 95% CI** | ***P*** |
| **Factor** |  |  |  |  |  |  |
| Male | 59/359 (16.4) | 81/569 (14.2) | 1.15, 0.85-1.57 | 0.361 |  |  |
| Age |  |  |  |  |  |  |
| <5 years | 1/22 (4.6) |  | 0.30, 0.04-2.06 | 0.221 |  |  |
| 5-9 years | 5/48 (10.4) |  | 0.69, 0.30-1.61 | 0.389 |  |  |
| 10-17 years | 16/76 (21.1) |  | 1.39, 0.87-2.22 | 0.163 |  |  |
| >17 years | 118/781 (15.1) |  | Reference |  |  |  |
| Person with secondary education | 86/533 (16.1) | 54/395 (13.7) | 1.18, 0.86-1.62 | 0.302 |  |  |
| Socio-economic status |  |  |  |  |  |  |
| High | 3/109 (2.8) |  | 0.12, 0.04-0.38 | <0.001 | 0.12, 0.04-0.40 | 0.001 |
| 2 | 25/187 (13.4) |  | 0.59, 0.37-0.93 | 0.024 | 0.59, 0.30-1.16 | 0.126 |
| 3 | 22/167 (13.2) |  | 0.58, 0.36-0.94 | 0.026 | 0.58, 0.30-1.13 | 0.110 |
| 4 | 40/191 (20.9) |  | 0.92, 0.62-1.37 | 0.678 | 0.92, 0.52-1.64 | 0.775 |
| Low | 36/158 (22.8) |  | Reference |  | Reference |  |
| Person with episode of malaria in previous year | 7/53 (13.2) | 133/875 (15.2) | 0.87, 0.43-1.76 | 0.697 |  |  |
| Rainy season at interview | 61/531 (11.5) | 79/397 (19.9) | 0.58, 0.42-0.79 | <0.001 | NS |  |

Abbreviations: CI: confidence interval, HH: household, NS: not significant, RR: risk ratio (prevalence ratio)

*Multivariate models were adjusted for clustering at the household level

Table S5B: Factors associated with the report of the use of a repellent at the individual level (survey) in Nadiad

|  | **Univariate analysis** | | | | **Multivariate analysis*** | |
| --- | --- | --- | --- | --- | --- | --- |
| **Nadiad: repellents** | **User with factor** | **User without factor** | **RR, 95% CI** | ***P*** | **RR, 95% CI** | ***P*** |
| **Factor or characteristic** |  |  |  |  |  |  |
| Male | 141/379 (37.2) | 176/417 (42.2) | 0.88, 0.74-1.05 | 0.151 |  |  |
| Age |  |  |  |  |  |  |
| <5 years | 11/20 (55.0) |  | 1.34, 0.89-2.01 | 0.164 | NS |  |
| 5-9 years | 15/57 (26.3) |  | 0.64, 0.41-0.99 | 0.048 |  |  |
| 10-17 years | 32/90 (35.6) |  | 0.86, 0.64-1.16 | 0.327 |  |  |
| >17 years | 259/629 (41.2) |  | Reference |  |  |  |
| Person with secondary education | 94/144 (65.3) | 223/651 (34.4) | 1.91, 1.62-2.24 | <0.001 | NS |  |
| Socio-economic status |  |  |  |  |  |  |
| High | 77/95 (81.1) |  | 4.45, 3.42-5.78 | <0.001 | 4.45, 2.90-6.82 | <0.001 |
| 2 | 76/97 (78.4) |  | 4.31, 3.30-5.61 | <0.001 | 4.30, 2.82-6.56 | <0.001 |
| 3 | 45/79 (57.0) |  | 3.13, 2.29-4.26 | <0.001 | 3.13, 1.93-5.08 | <0.001 |
| 4 | 44/181 (24.3) |  | 1.33, 0.94-1.90 | 0.110 | 1.33, 0.76-2.34 | 0.315 |
| Low | 53/291 (18.2) |  | Reference |  | Reference |  |
| Person with episode of malaria in previous year | 8/30 (26.7) | 309/766 (40.3) | 0.66, 0.36-1.20 | 0.176 |  |  |
| Rainy season at interview | 210/412 (51.0) | 107/384 (27.9) | 1.83, 1.52-2.20 | <0.001 | NS |  |
| **Nadiad: vaporizers** | **Users with factor** | **Users without factor** | **RR, 95% CI** | ***P*** | **RR, 95% CI** | ***P*** |
| **Factor** |  |  |  |  |  |  |
| Male | 44/379 (11.6) | 65/417 (15.6) | 0.74, 0.52-1.06 | 0.105 |  |  |
| Age |  |  |  |  |  |  |
| <5 years | 5/20 (25.0) |  | 1.73, 0.79-3.78 | 0.171 | NS |  |
| 5-9 years | 3/57 (5.3) |  | 0.36, 0.12-1.11 | 0.076 |  |  |
| 10-17 years | 10/90 (11.1) |  | 0.77, 0.42-1.42 | 0.400 |  |  |
| >17 years | 91/629 (14.5) |  | Reference |  |  |  |
| Person with secondary education | 48/144 (33.3) | 61/651 (9.4) | 3.56, 2.55-4.96 | <0.001 | NS |  |
| Socio-economic status |  |  |  |  |  |  |
| High | 31/95 (32.6) |  | 15.83, 6.81-36.77 | <0.001 | 15.83, 5.68-44.13 | <0.001 |
| 2 | 40/97 (41.2) |  | 20.00, 8.75-45.72 | <0.001 | 20.00, 7.43-53.87 | <0.001 |
| 3 | 11/79 (13.9) |  | 6.75, 2.58-17.69 | <0.001 | 6.75, 1.97-23.20 | 0.002 |
| 4 | 12/181 (6.6) |  | 3.22, 1.23-8.42 | 0.017 | 3.22, 1.03-10.00 | 0.044 |
| Low | 6/291 (2.1) |  | Reference |  | Reference |  |
| Person with episode of malaria in previous year | 2/30 (6.7) | 107/766 (14.0) | 0.48, 0.12-1.84 | 0.283 |  |  |
| Rainy season at interview | 77/412 (18.7) | 32/384 (8.3) | 2.24, 1.52-3.31 | <0.001 | NS |  |
| **Nadiad: coils** | **Users with factor** | **Users without factor** | **RR, 95% CI** | ***P*** | **RR, 95% CI** | ***P*** |
| **Factor** |  |  |  |  |  |  |
| Male | 97/379 (25.6) | 111/417 (26.6) | 0.96, 0.76-1.22 | 0.742 |  |  |
| Age |  |  |  |  |  |  |
| <5 years | 5/20 (25.0) |  | 0.93, 0.43-2.00 | 0.843 |  |  |
| 5-9 years | 11/57 (19.3) |  | 0.71, 0.41-1.23 | 0.227 |  |  |
| 10-17 years | 22/90 (24.4) |  | 0.90, 0.62-1.33 | 0.609 |  |  |
| >17 years | 170/629 (27.0) |  | Reference |  |  |  |
| Person with secondary education | 51/144 (35.4) | 157/651 (24.1) | 1.47, 1.13-1.90 | 0.004 | NS |  |
| Socio-economic status |  |  |  |  |  |  |
| High | 45/95 (47.4) |  | 4.18, 2.84-6.14 | <0.001 | 4.18, 2.13-8.21 | <0.001 |
| 2 | 41/97 (42.3) |  | 3.73, 2.51-5.54 | <0.001 | 3.73, 1.87-7.43 | <0.001 |
| 3 | 34/79 (43.0) |  | 3.80, 2.52-5.71 | <0.001 | 3.80, 1.91-7.56 | <0.001 |
| 4 | 39/181 (21.6) |  | 1.90, 1.24-2.91 | 0.003 | 1.90, 0.91-3.96 | 0.087 |
| Low | 33/291 (11.3) |  | Reference |  | Reference |  |
| Person with episode of malaria in previous year | 5/30 (16.7) | 203/766 (26.5) | 0.63, 0.28-1.41 | 0.261 |  |  |
| Rainy season at interview | 139/412 (33.7) | 69/384 (18.0) | 1.88, 1.46-2.42 | <0.001 | NS |  |

Abbreviations: CI: confidence interval, HH: household, NS: not significant, RR: risk ratio (prevalence ratio)

*Multivariate models are adjusted for clustering at the household level

Table S5C: Factors associated with the report of the use of a repellent at the individual level (survey) in Raurkela

|  |  | | **Univariate analysis** | | **Multivariate analysis*** | |
| --- | --- | --- | --- | --- | --- | --- |
| **Raurkela: repellents** | **Users among persons with factor** | **Users among persons without factor** | **RR, 95% CI** | ***P*** | **RR, 95% CI** | ***P*** |
| **Factor or characteristic** |  |  |  |  |  |  |
| Male | 227/692 (32.8) | 260/847 (30.7) | 1.07, 0.92-1.24 | 0.376 |  |  |
| Age |  |  |  |  |  |  |
| <5 years | 54/157 (34.4) |  | 1.07, 0.85-1.35 | 0.583 | NS |  |
| 5-9 years | 45/181 (24.9) |  | 0.77, 0.59-1.01 | 0.059 |  |  |
| 10-17 years | 61/186 (32.8) |  | 1.02, 0.81-1.27 | 0.876 |  |  |
| >17 years | 327/1015 (32.2) |  | Reference |  |  |  |
| Person with secondary education | 18/65 (27.7) | 469/1474 (31.8) | 0.87, 0.58-1.30 | 0.496 |  |  |
| Socio-economic status |  |  |  |  |  |  |
| High | 12/21 (57.1) |  | 1.61, 1.09-2.39 | 0.018 | 2.41, 1.46-3.97 | 0.001 |
| 2 | 12/44 (27.3) |  | 0.77, 0.47-1.27 | 0.303 | 1.03, 0.45-2.37 | 0.945 |
| 3 | 112/389 (28.8) |  | 0.81, 0.66-0.99 | 0.047 | 0.83, 0.58-1.19 | 0.316 |
| 4 | 211/693 (30.5) |  | 0.86, 0.72-1.02 | 0.087 | 0.79, 0.61-1.03 | 0.087 |
| Low | 484/1533 (31.6) |  | Reference |  | Reference |  |
| Person with episode of malaria in previous year | 121/399 (30.3) | 366/1140 (32.1) | 0.94, 0.80-1.12 | 0.513 |  |  |
| Rainy season at interview | 279/723 (38.6) | 208/816 (25.5) | 1.51, 1.30-1.76 | <0.001 | NS |  |
| ITN use | 257/469 (54.8) | 230/1070 (21.5) | 2.55, 2.21-2.93 | <0.001 | 2.71, 2.14-3.44 | <0.001 |
| **Raurkela: mats** | **Users with factor** | **Users without factor** | **RR, 95% CI** | ***P*** | **RR, 95% CI** | ***P*** |
| **Factor or characteristic** |  |  |  |  |  |  |
| Male | 193/692 (27.9) | 203/847 (24.0) | 1.16, 0.98-1.38 | 0.080 | NS |  |
| Age |  |  |  |  |  |  |
| <5 years | 47/157 (29.9) |  | 1.16, 0.90-1.51 | 0.254 | NS |  |
| 5-9 years | 38/181 (21.0) |  | 0.82. 0.60-1.10 | 0.187 |  |  |
| 10-17 years | 50/186 (26.9) |  | 1.05, 0.81-1.35 | 0.737 |  |  |
| >17 years | 261/1015 (25.7) |  | Reference |  |  |  |
| Person with secondary education | 7/65 (10.8) | 389/1474 (26.4) | 0.41, 0.20-0.83 | 0.013 | 0.40, 0.20-0.81 | 0.010 |
| Socio-economic status |  |  |  |  |  |  |
| High | 0/21 (0.0) |  |  |  | NS |  |
| 2 | 3/44 (6.8) |  | 0.21, 0.07-0.64 | 0.006 |  |  |
| 3 | 82/389 (21.1) |  | 0.66, 0.52-0.84 | 0.001 |  |  |
| 4 | 188/693 (27.1) |  | 0.85, 0.70-1.03 | 0.097 |  |  |
| Low | 123/386 (31.9) |  | Reference |  |  |  |
| Person with episode of malaria in previous year | 93/399 (23.3) | 303/1140 (26.6) | 0.88, 0.72-1.07 | 0.204 |  |  |
| Rainy season at interview | 225/723 (31.1) | 171/816 (21.0) | 1.49, 1.25-1.76 | <0.001 | NS |  |
| ITN use | 247/469 (52.7) | 149/1070 (13.9) | 3.78, 3.18-4.49 | <0.001 | 3.79, 2.91-4.92 | <0.001 |
| **Raurkela: coils** | **Users with factor** | **Users without factor** | **RR, 95% CI** | ***P*** | **RR, 95% CI** | ***P*** |
| **Factor or characteristic** |  |  |  |  |  |  |
| Male | 29/692 (4.2) | 54/847 (6.4) | 0.66, 0.42-1.02 | 0.062 | NS |  |
| Age |  |  |  |  |  |  |
| <5 years | 7/157 (4.5) |  | 0.75, 0.35-1.62 | 0.470 | NS |  |
| 5-9 years | 5/181 (2.8) |  | 0.46, 0.19-1.15 | 0.097 |  |  |
| 10-17 years | 11/186 (5.9) |  | 1.00, 0.54-1.87 | 0.999 |  |  |
| >17 years | 60/1015 (5.9) |  | Reference |  |  |  |
| Person with secondary education | 12/65 (18.5) | 71/1474 (4.8) | 3.83, 2.19-6.70 | <0.001 | 2.08, 0.96-4.51 | 0.064 |
| Socio-economic status |  |  |  |  |  |  |
| High | 11/21 (52.4) |  | 16.84, 8.45-33.60 | <0.001 | 21.95, 8.12-59.32 | <0.001 |
| 2 | 10/44 (22.7) |  | 7.31, 3.35-15.93 | <0.001 | 5.18, 1.40-19.16 | 0.014 |
| 3 | 25/389 (6.4) |  | 2.07, 1.05-4.06 | 0.035 | 1.61, 0.59-4.37 | 0.348 |
| 4 | 22/693 (3.2) |  | 1.02, 0.51-2.04 | 0.953 | 0.95, 0.38-2.40 | 0.915 |
| Low | 12/386 (3.1) |  | Reference |  | Reference |  |
| Person with episode of malaria in previous year | 25/399 (6.3) | 58/1140 (5.1) | 1.23, 0.78-1.94 | 0.370 |  |  |
| Rainy season at interview | 48/723 (6.6) | 35/816 (4.3) | 1.55, 1.02-2.37 | 0.043 | 3.27, 1.55-6.89 | 0.002 |
| ITN use | 7/469 (1.5) | 76/1070 (7.1) | 0.21, 0.10-0.45 | <0.001 | 0.20, 0.06-0.66 | 0.008 |
| **Raurkela: ITNs** | **Users with factor** | **Users without factor** | **RR, 95% CI** | ***P*** | **RR, 95% CI** | ***P*** |
| **Factor or characteristic** |  |  |  |  |  |  |
| Male | 226/692 (32.7) | 243/847 (28.7) | 1.14, 0.98-1.32 | 0.092 | NS |  |
| Age |  |  |  |  |  |  |
| <5 years | 40/157 (25.5) |  | 0.85, 0.64-1.13 | 0.254 | 0.83, 0.62-1.11 | 0.202 |
| 5-9 years | 53/181 (29.3) |  | 0.97, 0.76-1.25 | 0.836 | 1.10, 0.87-1.39 | 0.413 |
| 10-17 years | 71/186 (38.2) |  | 1.27, 1.03-1.56 | 0.023 | 1.29, 1.07-1.56 | 0.008 |
| >17 years | 305/1015 (30.1) |  | Reference |  | Reference |  |
| Person with secondary education | 19/65 (29.2) | 450/1474 (30.5) | 0.96, 0.65-1.41 | 0.825 | NS |  |
| Socio-economic status |  |  |  |  |  |  |
| High | 0/21 (0.0) |  |  |  |  |  |
| 2 | 3/44 (6.8) |  | 0.23, 0.08-0.71 | 0.010 | NS |  |
| 3 | 104/389 (26.7) |  | 0.92, 0.73-1.16 | 0.479 |  |  |
| 4 | 249/693 (35.9) |  | 1.24, 1.03-1.49 | 0.024 |  |  |
| Low | 112/386 (29.0) |  | Reference |  |  |  |
| Person with episode of malaria in previous year | 136/399 (34.1) | 333/1140 (29.2) | 1.17, 0.99-1.37 | 0.065 | 1.27, 1.08-1.49 | 0.004 |
| Rainy season at interview | 334/723 (46.2) | 135/816 (16.5) | 2.79, 2.35-3.32 | <0.001 | 2.48, 1.89-3.25 | <0.001 |
| Use of mats | 247/396 (62.4) | 222/1143 (19.4) | 3.21, 2.79-3.70 | <0.001 | 2.93, 2.32-3.71 | <0.001 |
| Use of coils | 7/83 (8.4) | 462/1456 (31.7) | 0.27, 0.13-0.54 | <0.001 | NS |  |

Abbreviations: CI: confidence interval, HH: household, NS: not significant, RR: risk ratio (prevalence ratio)

*Multivariate models are adjusted for clustering at the household level

Table S6A: Factors associated with the report of the use of a repellent among clinic patients in Chennai

|  |  | | **Univariate analysis** | | **Multivariate analysis** | |
| --- | --- | --- | --- | --- | --- | --- |
| **Chennai: repellents** | **User with factor** | **User without factor** | **RR, 95% CI** | ***P*** | **RR, 95% CI** | ***P*** |
| **Factor** |  |  |  |  |  |  |
| Male | 248/717 (34.6) | 107/337 (31.8) | 1.09, 0.90-1.31 | 0.367 |  |  |
| Age |  |  |  |  |  |  |
| <5 years | 4/10 (40.0) |  | 1.17, 0.55-2.52 | 0.681 |  |  |
| 5-9 years | 9/28 (32.1) |  | 0.94, 0.55-1.63 | 0.835 |  |  |
| 10-17 years | 32/106 (30.2) |  | 0.89, 0.65-1.20 | 0.435 |  |  |
| >17 years | 310/910 (34.1) |  | Reference |  |  |  |
| Person with secondary education | 245/642 (38.2) | 109/409 (26.7) | 1.43, 1.19-1.73 | <0.001 | 1.36, 1.13-1.65 | 0.001 |
| Occupation |  |  |  |  |  |  |
| None | 102/309 (33.0) |  | 0.89, 0.73-1.08 | 0.251 | 0.90, 0.74-1.09 | 0.286 |
| Daily wage/labor | 18/92 (19.6) |  | 0.53, 0.34-0.81 | 0.004 | 0.58, 0.37-0.89 | 0.012 |
| Trade (self-employed) | 51/161 (31.7) |  | 0.86, 0.66-1.10 | 0.229 | 0.87, 0.68-1.12 | 0.289 |
| Salaried | 180/486 (37.0) |  | Reference |  | Reference |  |
| Person with episode of malaria in previous year | 61/175 (34.9) | 294/879 (33.5) | 1.04, 0.83-1.30 | 0.717 |  |  |
| Rainy season at interview | 205/640 (32.0) | 150/414 (36.2) | 0.88, 0.75-1.05 | 0.157 |  |  |
| **Chennai: vaporizers** | **Users with factor** | **Users without factor** | **RR, 95% CI** | ***P*** | **RR, 95% CI** | ***P*** |
| **Factor** |  |  |  |  |  |  |
| Male | 159/717 (22.2) | 68/337 (20.2) | 1.10, 0.85-1.42 | 0.464 |  |  |
| Age |  |  |  |  |  |  |
| <5 years | 3/10 (30.0) |  | 1.38, 0.53-3.58 | 0.510 |  |  |
| 5-9 years | 9/28 (32.1) |  | 1.48, 0.85-2.57 | 0.166 |  |  |
| 10-17 years | 17/106 (16.0) |  | 0.74, 0.47-1.16 | 0.187 |  |  |
| >17 years | 198/910 (21.8) |  | Reference |  |  |  |
| Person with secondary education | 159/642 (24.8) | 68/409 (16.6) | 1.49, 1.15-1.92 | 0.002 | 1.41, 1.09-1.82 | 0.009 |
| Occupation |  |  |  |  |  |  |
| None | 63/309 (20.4) |  | 0.88, 0.67-1.15 | 0.346 | 0.86, 0.65-1.13 | 0.266 |
| Daily wage/labor | 11/92 (12.0) |  | 0.51, 0.29-0.92 | 0.024 | 0.55, 0.31-0.99 | 0.045 |
| Trade (self-employed) | 37/161 (23.0) |  | 0.99, 0.71-1.37 | 0.944 | 0.98, 0.71-1.36 | 0.903 |
| Salaried | 113/486 (23.3) |  | Reference |  | Reference |  |
| Person with episode of malaria in previous year | 39/175 (22.3) | 188/879 (21.4) | 1.04, 0.77-1.41 | 0.791 |  |  |
| Rainy season at interview | 123/640 (19.2) | 104/414 (25.1) | 0.77, 0.61-0.96 | 0.022 | 0.76, 0.60-0.95 | 0.019 |
| **Chennai: coils** | **Users with factor** | **Users without factor** | **RR, 95% CI** | ***P*** | **RR, 95% CI** | ***P*** |
| **Factor** |  |  |  |  |  |  |
| Male | 95/717 (13.3) | 44/337 (13.1) | 1.01, 0.73-1.42 | 0.931 |  |  |
| Age |  |  |  |  |  |  |
| <5 years | 1/9 (10.0) |  | 0.75, 0.12-4.82 | 0.758 |  |  |
| 5-9 years | 0/28 (0.0) |  |  |  |  |  |
| 10-17 years | 16/106 (15.1) |  | 1.13, 0.70-1.82 | 0.629 |  |  |
| >17 years | 122/910 (13.4) |  | Reference |  |  |  |
| Person with secondary education | 92/642 (14.3) | 46/409 (11.3) | 1.27, 0.91-1.77 | 0.152 |  |  |
| Occupation |  |  |  |  |  |  |
| None | 42/309 (13.6) |  | 0.90, 0.64-1.29 | 0.578 | 0.90, 0.64-1.29 | 0.578 |
| Daily wage/labor | 6/92 (6.5) |  | 0.43, 0.19-0.97 | 0.041 | 0.43, 0.19-0.97 | 0.041 |
| Trade (self-employed) | 17/161 (10.6) |  | 0.70, 0.43-1.16 | 0.164 | 0.70, 0.43-1.16 | 0.164 |
| Salaried | 73/486 (15.0) |  | Reference |  | Reference |  |
| Person with episode of malaria in previous year | 24/175 (13.7) | 115/879 (13.1) | 1.05, 0.70-1.58 | 0.821 |  |  |
| Rainy season at interview | 89/640 (13.9) | 50/414 (12.1) | 1.15, 0.83-1.59 | 0.393 |  |  |

Abbreviations: CI: confidence interval, HH: household, NS: not significant, RR: risk ratio (prevalence ratio)

Table S6B: Factors associated with the report of the use of a repellent among clinic patients in Nadiad

|  |  | | **Univariate analysis** | | **Multivariate analysis** | |
| --- | --- | --- | --- | --- | --- | --- |
| **Nadiad: repellents** | **User with factor** | **User without factor** | **RR, 95% CI** | ***P*** | **RR, 95% CI** | ***P*** |
| **Factor** |  |  |  |  |  |  |
| Male | 204/414 (49.3) | 140/271 (51.7) | 0.95, 0.82-1.11 | 0.540 |  |  |
| Age |  |  |  |  |  |  |
| <5 years | 16/26 (61.5) |  | 1.31, 0.95-1.80 | 0.097 | 1.29, 0.75-2.20 | 0.355 |
| 5-9 years | 63/89 (70.8) |  | 1.51, 1.28-1.78 | <0.001 | 1.61, 1.16-2.24 | 0.004 |
| 10-17 years | 49/110 (44.6) |  | 0.95, 0.75-1.19 | 0.653 | 0.99, 0.71-1.40 | 0.970 |
| >17 years | 216/460 (47.0) |  | Reference |  | Reference |  |
| Person with secondary education | 86/146 (58.9) | 258/539 (47.9) | 1.23, 1.05-1.45 | 0.012 | 1.33, 1.01-1.73 | 0.039 |
| Occupation |  |  |  |  |  |  |
| None | 221/420 (52.6) |  | 0.87, 0.72-1.05 | 0.152 | 0.76, 0.54-1.07 | 0.112 |
| Daily wage/labor | 42/120 (35.0) |  | 0.58, 0.43-0.78 | <0.001 | 0.59, 0.39-0.90 | 0.013 |
| Trade (self-employed) | 26/54 (48.2) |  | 0.80, 0.58-1.10 | 0.168 | 0.80, 0.50-1.29 | 0.363 |
| Salaried | 55/91 (60.4) |  | Reference |  | Reference |  |
| Person with episode of malaria in previous year | 29/50 (58.0) | 315/635 (49.6) | 1.17, 0.91-1.50 | 0.218 | NS |  |
| Rainy season at interview | 37/145 (25.5) | 307/540 (56.9) | 0.45, 0.34-0.60 | <0.001 | 0.43, 0.31-0.61 | <0.001 |
| **Nadiad: vaporizers** | **Users with factor** | **Users without factor** | **RR, 95% CI** | ***P*** | **RR, 95% CI** | ***P*** |
| **Factor** |  |  |  |  |  |  |
| Male | 67/414 (16.2) | 45/271 (16.6) | 0.97, 0.69-1.38 | 0.884 |  |  |
| Age |  |  |  |  |  |  |
| <5 years | 8/26 (30.8) |  | 2.08, 1.12-3.86 | 0.020 | 2.21, 1.12-4.33 | 0.021 |
| 5-9 years | 17/89 (19.1) |  | 1.29, 0.80-2.09 | 0.296 | 1.51, 0.87-2.63 | 0.140 |
| 10-17 years | 19/110 (17.3) |  | 1.17, 0.73-1.86 | 0.511 | 1.22, 0.74-2.01 | 0.438 |
| >17 years | 68/460 (14.8) |  | Reference |  | Reference |  |
| Person with secondary education | 39/146 (26.7) | 73/539 (13.5) | 1.97, 1.40-2.78 | <0.001 | 2.03, 1.39-2.98 | <0.001 |
| Occupation |  |  |  |  |  |  |
| None | 75/420 (17.9) |  | 0.71, 0.47-1.06 | 0.096 | 0.68, 0.43-1.09 | 0.107 |
| Daily wage/labor | 5/120 (4.2) |  | 0.16, 0.07-0.42 | <0.001 | 0.20, 0.08-0.51 | 0.001 |
| Trade (self-employed) | 9/54 (16.7) |  | 0.66, 0.33-1.32 | 0.239 | 0.73, 0.37-1.42 | 0.350 |
| Salaried | 23/91 (25.3) |  | Reference |  | Reference |  |
| Person with episode of malaria in previous year | 10/50 (20.0) | 102/635 (16.1) | 1.25, 0.70-2.23 | 0.461 |  |  |
| Rainy season at interview | 10/145 (6.9) | 102/540 (18.9) | 0.37, 0.20-0.68 | 0.002 | 2.03, 1.39-2.98 | <0.001 |
| **Nadiad: coils** | **Users with factor** | **Users without factor** | **RR, 95% CI** | ***P*** | **RR, 95% CI** | ***P*** |
| **Factor** |  |  |  |  |  |  |
| Male | 114/414 (27.5) | 76/271 (28.0) | 0.98, 0.77-1.26 | 0.884 |  |  |
| Age |  |  |  |  |  |  |
| <5 years | 8/26 (30.8) |  | 1.14, 0.63-2.07 | 0.663 | 1.02, 0.56-1.84 | 0.960 |
| 5-9 years | 38/89 (42.7) |  | 1.58, 1.19-2.10 | 0.001 | 1.52, 1.16-2.01 | 0.003 |
| 10-17 years | 20/110 (18.2) |  | 0.67, 0.44-1.03 | 0.069 | 0.68, 0.45-1.04 | 0.073 |
| >17 years | 124/460 (27.0) |  | Reference |  | Reference |  |
| Person with secondary education | 32/146 (21.9) | 158/539 (29.3) | 0.75, 0.54-1.04 | 0.087 | NS |  |
| Occupation |  |  |  |  |  |  |
| None | 117/420 (27.9) |  | 0.94, 0.66-1.33 | 0.725 |  |  |
| Daily wage/labor | 33/120 (27.5) |  | 0.93, 0.60-1.42 | 0.729 |  |  |
| Trade (self-employed) | 13/54 (24.1) |  | 0.81, 0.46-1.43 | 0.472 |  |  |
| Salaried | 27/91 (29.7) |  | Reference |  |  |  |
| Person with episode of malaria in previous year | 20/50 (40.0) | 170/635 (26.8) | 1.49, 1.04-2.15 | 0.030 | NS |  |
| Rainy season at interview | 18/145 (12.4) | 172/540 (31.9) | 0.39, 0.25-0.61 | <0.001 | 0.40, 0.26-0.63 | <0.001 |

Abbreviations: CI: confidence interval, HH: household, NS: not significant, RR: risk ratio (prevalence ratio)

Table S6C: Factors associated with the report of the use of a repellent among clinic patients in Raurkela

|  | **Univariate analysis** | | | | **Multivariate analysis** | |
| --- | --- | --- | --- | --- | --- | --- |
| **Raurkela: repellents** | **User with factor** | **User without factor** | **RR, 95% CI** | ***P*** | **RR, 95% CI** | ***P*** |
| **Factor or characteristics** |  |  |  |  |  |  |
| Male | 831/1076 (77.20 | 630/799 (78.9) | 0.98, 0.93-1.03 | 0.401 |  |  |
| Age |  |  |  |  |  |  |
| <5 years | 124/162 (76.5) |  | 0.99, 0.91-1.08 | 0.845 |  |  |
| 5-9 years | 150/184 (81.5) |  | 1.06, 0.98-1.14 | 0.159 |  |  |
| 10-17 years | 220/277 (79.4) |  | 1.03, 0.96-1.10 | 0.415 |  |  |
| >17 years | 967/1252 (77.2) |  | Reference |  |  |  |
| Person with secondary education | 592/737 (80.3) | 869/1138 (76.4) | 1.05, 1.00-1.10 | 0.040 | NS |  |
| Occupation |  |  |  |  |  |  |
| None | 1020/1292 (79.0) |  | 1.01, 0.95-1.07 | 0.785 | 1.01, 0.95-1.07 | 0.785 |
| Daily wage/labor | 55/93 (59.1) |  | 0.76, 0.63-0.90 | 0.002 | 0.76, 0.63-0.90 | 0.002 |
| Trade (self-employed) | 83/103 (80.6) |  | 1.03, 0.92-1.15 | 0.602 | 1.03, 0.92-1.15 | 0.602 |
| Salaried | 303/387 (78.3) |  | Reference |  | Reference |  |
| Person with episode of malaria in previous year | 93/125 (74.4) | 1368/1750 (78.2) | 0.95, 0.86-1.06 | 0.360 |  |  |
| Rainy season at interview | 780/1005 (77.6) | 681/870 (78.3) | 0.99, 0.94-1.04 | 0.729 |  |  |
| **Raurkela: vaporizers** | **Users with factor** | **Users without factor** | **RR, 95% CI** | ***P*** | **RR, 95% CI** | ***P*** |
| **Factor or characteristic** |  |  |  |  |  |  |
| Male | 417/1076 (38.8) | 310/799 (38.8) | 1.00, 0.89-1.12 | 0.985 |  |  |
| Age |  |  |  |  |  |  |
| <5 years | 80/162 (49.4) |  | 1.34, 1.13-1.59 | 0.001 | 1.42, 1.16-1.73 | 0.001 |
| 5-9 years | 82/184 (44.6) |  | 1.21, 1.01-1.44 | 0.034 | 1.32, 1.07-1.61 | 0.008 |
| 10-17 years | 104/277 (37.6) |  | 1.02, 0.86-1.21 | 0.821 | 1.06, 0.88-1.26 | 0.557 |
| >17 years | 461/1252 (36.8) |  | Reference |  | Reference |  |
| Person with secondary education | 319/737 (43.3) | 408/1138 (35.9) | 1.21, 1.08-1.35 | 0.001 | 1.34, 1.16-1.54 | <0.001 |
| Occupation |  |  |  |  |  |  |
| None | 527/1292 (40.8) |  | 1.04, 0.90-1.19 | 0.597 | 1.05, 0.90-1.22 | 0.561 |
| Daily wage/labor | 9/93 (9.7) |  | 0.25, 0.13-0.46 | <0.001 | 0.29, 0.15-0.55 | <0.001 |
| Trade (self-employed) | 39/103 (37.9) |  | 0.96, 0.73-1.27 | 0.795 | 1.08, 0.82-1.42 | 0.574 |
| Salaried | 152/387 (39.3) |  | Reference |  | Reference |  |
| Person with episode of malaria in previous year | 32/125 (25.6) | 695/1750 (39.7) | 0.64, 0.48-0.87 | 0.005 | 0.72, 0.53-0.97 | 0.033 |
| Rainy season at interview | 353/1005 (35.1) | 374/870 (43.0) | 0.82, 0.73-0.92 | <0.001 | 0.81, 0.72-0.90 | <0.001 |
| **Raurkela: coils** | **Users with factor** | **Users without factor** | **RR, 95% CI** | ***P*** | **RR, 95% CI** | ***P*** |
| **Factor or characteristic** |  |  |  |  |  |  |
| Male | 450/1076 (41.8) | 348/799 (43.6) | 0.96, 0.86-1.07 | 0.452 |  |  |
| Age |  |  |  |  |  |  |
| <5 years | 54/162 (33.3) |  | 0.77, 0.61-0.96 | 0.023 | 0.79, 0.63-0.99 | 0.046 |
| 5-9 years | 72/184 (39.1) |  | 0.90, 0.75-1.09 | 0.291 | 0.91, 0.76-1.11 | 0.355 |
| 10-17 years | 129/277 (46.6) |  | 1.07, 0.93-1.24 | 0.323 | 1.06, 0.92-1.22 | 0.403 |
| >17 years | 543/1252 (43.4) |  | Reference |  | Reference |  |
| Person with secondary education | 304/737 (41.3) | 494/1138 (43.4) | 0.95, 0.85-1.06 | 0.357 |  |  |
| Occupation |  |  |  |  |  |  |
| None | 550/1292 (42.6) |  | 1.04, 0.91-1.19 | 0.607 |  |  |
| Daily wage/labor | 43/93 (46.2) |  | 1.13, 0.88-1.44 | 0.354 |  |  |
| Trade (self-employed) | 46/103 (44.7) |  | 1.09, 0.85-1.39 | 0.506 |  |  |
| Salaried | 159/387 (41.1) |  | Reference |  |  |  |
| Person with episode of malaria in previous year | 63/125 (50.4) | 735/1750 (42.0) | 1.20, 1.00-1.44 | 0.050 | NS |  |
| Rainy season at interview | 465/1005 (46.3) | 333/870 (38.3) | 1.21, 1.09-1.35 | 0.001 | 1.19, 1.07-1.32 | 0.002 |

Abbreviations: CI: confidence interval, HH: household, NS: not significant, RR: risk ratio (prevalence ratio)

Table S7: Malaria by use of repellent, study location and type of study

|  | **Survey*** | | | | **Clinic** | | | |
| --- | --- | --- | --- | --- | --- | --- | --- | --- |
|  | **Malaria among users** | **Malaria among non-users** | **RR, 95% CI** | ***P*** | **Malaria among users** | **Malaria among non-users** | **RR, 95% CI** | ***P*** |
| **Any Repellent** | | |  |  |  |  |  |  |
| Chennai | 4/422 (1.0) | 4/506 (0.8) | 1.20, 0.30-4.80 | 0.797 | 50/355 (14.1) | 136/699 (19.5) | 0.72, 0.54-0.98 | 0.034 |
| Nadiad | 16/317 (5.1) | 41/479 (8.6) | 0.63, 0.35-1.14 | 0.124 | 28/344 (8.1) | 43/341 (12.6) | 0.65, 0.41-1.01 | 0.058 |
| Raurkela | 47/487 (9.7) | 80/1052 (7.6) | 1.27, 0.81-1.98 | 0.297 | 41/1461 (2.8) | 22/414 (5.3) | 0.53, 0.32-0.88 | 0.013 |
| **Vaporizer** |  |  |  |  |  |  |  |  |
| Chennai | 1/310 (0.3) | 7/618 (1.1) | 0.28, 0.03-2.32 | 0.240 | 34/277 (15.0) | 152/827 (18.4) | 0.81, 0.58-1.15 | 0.240 |
| Nadiad | 10/109 (9.2) | 47/687 (6.8) | 1.44, 0.73-2.85 | 0.296 | 7/112 (6.3) | 64/573 (11.2) | 0.56, 0.26-1.19 | 0.131 |
| Raurkela |  |  | Not applicable |  | 12/727 (1.7) | 51/1148 (4.4) | 0.37, 0.20-0.69 | 0.002 |
| **Coil** |  |  |  |  |  |  |  |  |
| Chennai | 3/140 (2.1) | 5/788 (0.6) | 3.40, 0.81-14.20 | 0.094 | 19/139 (13.7) | 167/915 (18.3) | 0.75, 0.48-1.16 | 0.198 |
| Nadiad | 7/208 (3.4) | 50/588 (8.5) | 0.42, 0.19-0.91 | 0.027 | 18/190 (9.5) | 53/495 (10.7) | 0.88, 0.53-1.47 | 0.637 |
| Raurkela | 6/83 (4.7) | 121/1456(8.3) | 0.87, 0.39-1.92 | 0.730 | 29/798 (3.6) | 34/1077 (3.2) | 1.16, 0.71-1.87 | 0.571 |
| **Mat** |  |  |  |  |  |  |  |  |
| Raurkela | 41/396 (10.4) | 86/1143 (7.5) | 1.37, 0.87-2.17 | 0.171 |  | Not applicable |  |  |
| **ITN** |  |  |  |  |  |  |  |  |
| Raurkela | 61/469 (13.0) | 66/1070 (6.2) | 2.11, 1.39-3.21 | <0.001 |  | Not applicable |  |  |

Abbreviations: CI: confidence interval, HH: household, NS: not significant, RR: risk ratio (prevalence ratio). *P*-value obtained from generalized linear univariate regression model.

*Survey data adjusted for clustering at the household level

References

1. Snehalatha KS, Ramaiah KD, Vijay Kumar KN, Das PK. The mosquito problem and type and costs of personal protection measures used in rural and urban communities in Pondicherry region, South India. Acta Trop. 2003;88:3-9.

2. Babu BV, Mishra S, Mishra S, Swain BK. Personal-protection measures against mosquitoes: a study of practices and costs in a district, in the Indian state of Orissa, where malaria and lymphatic filariasis are co-endemic. Ann Trop Med Parasitol. 2007;101:601-9.

3. Singh RK, Haq S, Dhiman RC. Studies on knowledge, attitude and practices in malaria endemic tribal areas of Bihar and Jharkhand, India. Journal of Tropical Diseases. 2013 1:1000110.

4. Kowli SS, Attar H. Efficacy of prophylactic measures for malaria. Review of Global Medicine and Health Care Research. 2010;1:195-214.

5. Dhawan G, Joseph N, Pekow PS, Rogers CA, Poudel KC, Bulzacchelli MT. Malaria-related knowledge and prevention practices in four neighbourhoods in and around Mumbai, India: a cross-sectional study. Malar J. 2014;13:303.

6. Vala MC, Patel UV, Joshi NB, Zalavadiya DD, Viramgami AP, Sharma S. Knowledge and practice regarding malaria among people of urban and rural areas of Rajkot District, Gujarat, India. Int J Res Med. 2013;2:38-42.

7. Chitra GA, Kaur P, Bhatnagar T, Manickam P, Murhekar MV. High prevalence of household pesticides and their unsafe use in rural South India. Int J Occup Med Environ Health. 2013;26:275-82.

8. Bhattacharyya H. Knowledge, beliefs, and practices regarding malaria in urban setting of East Khasi Hills district, Meghalaya. International Journal of Medical Science and Public Health. 2015;4:1042-5.
